# CSF1R antagonism limits local restimulation of antiviral CD8^+^ T cells during viral encephalitis

**DOI:** 10.1186/s12974-019-1397-4

**Published:** 2019-01-31

**Authors:** Kristen E. Funk, Robyn S. Klein

**Affiliations:** 1 Department of Internal Medicine, Division of Infectious Diseases, Washington University School of Medicine, Saint Louis, MO 63110 USA; 2 Department of Pathology and Immunology, Washington University School of Medicine, Saint Louis, MO 63110 USA; 3 Department of Neurosciences, Washington University School of Medicine, Saint Louis, MO 63110 USA

**Keywords:** West Nile virus, Microglia, Antiviral T cells, Viral encephalitis, Colony-stimulating factor 1 receptor, PLX5622

## Abstract

**Background:**

Microglia are resident macrophages of the central nervous system (CNS) locally maintained through colony-stimulating factor 1 receptor (CSF1R) signaling. Microglial depletion via CSF1R inactivation improves cognition in mouse models of neuroinflammation, but limits virologic control in the CNS of mouse models of neurotropic infections by unknown mechanisms. We hypothesize that CSF1R plays a critical role in myeloid cell responses that restrict viral replication and locally restimulate recruited antiviral T cells within the CNS.

**Methods:**

The impact of CSF1R signaling during West Nile virus infection was assessed in vivo using a mouse model of neurotropic infection. Pharmacological inactivation of CSF1R was achieved using PLX5622 prior to infection with virulent or attenuated strains of West Nile virus (WNV), an emerging neuropathogen. The subsequent effect of CSF1R antagonism on virologic control was assessed by measuring mortality and viral titers in the CNS and peripheral organs. Immune responses were assessed by flow cytometric-based phenotypic analyses of both peripheral and CNS immune cells.

**Results:**

Mice treated with CSF1R antagonist prior to infection exhibited higher susceptibility to lethal WNV infection and lack of virologic control in both the CNS and periphery. CSFR1 antagonism reduced B7 co-stimulatory signals on peripheral and CNS antigen-presenting cells (APCs) by depleting CNS cellular sources, which limited local reactivation of CNS-infiltrating virus-specific T cells and reduced viral clearance.

**Conclusions:**

Our results demonstrate the impact of CSF1R antagonism on APC activation in the CNS and periphery and the importance of microglia in orchestrating the CNS immune response following neurotropic viral infection. These data will be an important consideration when assessing the benefit of CSF1R antagonism, which has been investigated as a therapeutic for neurodegenerative conditions, in which neuroinflammation is a contributing factor.

**Electronic supplementary material:**

The online version of this article (10.1186/s12974-019-1397-4) contains supplementary material, which is available to authorized users.

## Background

West Nile virus (WNV) is an emerging human pathogen that causes seasonal outbreaks through the Western hemisphere since its introduction in 1999 [1]. A member of the *Flavivirus* genus, WNV is an enveloped, single-stranded positive sense RNA virus that cycles between birds and mosquitos. Symptoms of WNV infection can range from a relatively mild flu-like illness, West Nile fever, to severe neuroinvasive disease. After peripheral infection, WNV replicates within lymphoid tissues before entering the CNS, where it targets neurons in the cerebellum, brainstem, and cerebral cortex [2, 3]. In 2017, 2002 symptomatic WNV cases were reported to the CDC, and of those, 1339 (67%) were classified as neuroinvasive [4]. Prolonged neurological deficits, which include motor functions, verbal learning and memory, and executive functions, are a significant problem for survivors of WNV infection and appear to manifest even in cases where neuroinvasion is absent [5–7]. Recent work indicates that persistent phagocytic microglia contribute to prolonged cognitive dysfunction following WNV encephalitis [8], and thus, limiting microglial activation may alleviate the associated neurological sequelae.

Microglia are the resident immune cells in the CNS, originating from yolk sac progenitor cells that seed the brain early in development [9]. In addition to a multitude of homeostatic functions throughout development and healthy aging [10, 11], microglia participate in both innate and adaptive immune responses through recognition of pathogen-associated molecular patterns and damage-associated molecular patterns [12], which stimulate morphological changes [13, 14] and increase production of reactive oxidative species [15, 16] and cytokines/chemokines [17, 18]. During neurotropic virus infection, virally infected cells secrete chemokines including CCL2, CCL5, and CXCL10, which facilitate immune cells crossing the blood brain barrier (BBB) [19–21]. Mononuclear cell infiltration into the CNS is a hallmark of WNV encephalitis, and monocytes and T cells are critical for CNS virologic control. Elimination of monocytes using a liposome-encapsulated drug dichloromethylene diphosphonate or by genetic deletion of *CCR2* increases mortality in mice infected with a neurotropic strain of WNV [22, 23]. However, monocytes may also contribute to dissemination of virus to the CNS [24, 25], and inhibition of monocyte infiltration may also protect against nitric oxide-mediated immunopathology [26]. CD4^+^ T cells produce antiviral molecules, such as interferon γ (IFNγ) and IL-2, which promotes antiviral humoral immunity and sustains WNV-specific CD8^+^ T cell responses, enabling viral clearance in all organs [27, 28]. CD8^+^ T cells contribute to viral clearance and recovery from WNV infection via both cytopathic and noncytopathic mechanisms [29–32]. Mice lacking either CD8^+^ T cells or MHC-I molecules have higher CNS viral burdens, increased mortality after infection, and prolonged viral persistence, suggesting that cell-mediated immunity controls virus within the CNS [32].

CSF1 receptor (CSF1R) signaling is essential for the development of many mononuclear phagocytes including microglia. Tissue-resident macrophages, such as microglia, fail to develop in *Csf1r*^−/−^ mice [9], but in contrast to many tissue macrophages, adult microglia can still form in *Csf1*^*op/op*^ mice, which carry a natural null mutation in the *Csf1* gene [33]. This is due to an alternative CSF1R ligand, IL-34, which is highly expressed by neurons [34, 35]. Similar to *Csf1*^*op/op*^ mice, *IL-34*^*−/−*^ mice develop fewer microglia but do not lack peripheral macrophages [36]. These mice are more susceptible to lethal neurotropic WNV infection, which is associated with increased neuronal apoptosis without increased viral replication [36]. In order to temporally control microglial depletion, pharmacologic agents that antagonize CSF1R have been developed, including antagonist PLX5622, which depletes microglia in as little as 3 days and may be sustained for at least 6 weeks [37]. These agents are being investigated as therapeutics in models of neurodegenerative diseases in which microglial activation is considered a contributing factor, such as traumatic brain injury [38, 39], experimental autoimmune encephalomyelitis [40], and Alzheimer’s disease [41]. Here, we used PLX5622 in a model of WNV encephalitis to examine the effect of CSF1R antagonism in the setting of neurotropic viral infection. Our results show that mice treated with PLX5622 are more susceptible to lethal WNV infection, which is associated with increased CNS viral burden and neuronal apoptosis. PLX5622 also reduced expression of proinflammatory cytokines and B7 co-stimulatory molecules within the infected CNS by depleting cellular sources, including resident microglia and infiltrating macrophages, as well as decreasing expression on peripheral antigen-presenting cells (APCs). The loss of APCs in the CNS resulted in limited local reactivation of recruited antiviral CD8^+^ T cells, which are required for virologic control in the CNS. Together, these data demonstrate the impact of CSF1R antagonism on APC activation during viral infection in both the CNS and periphery and highlight the importance of microglia in orchestrating cell-mediated antiviral immunity during neurotropic infection.

## Methods

### Ethics statement

All experiments were performed in strict compliance with the recommendations in the Guide for the Care and Use of Laboratory Animals of the National Institutes of Health and according to the international Guiding Principles for Biomedical Research Involving Animals. The protocol was approved by the Washington University School of Medicine in St Louis Animal Safety Committee (#20170064).

### Viruses

WNV-NY is strain 3000.0259, isolated in New York in 2000 [42] and passaged twice in C6/36 *Aedes albopictus* cells to generate an insect cell-derived stock for f.p. inoculations. For i.c. inoculations of WNV-NY, virus strain 3000.0259 was passaged once in Vero cells to generate mammalian cell-derived stock. Attenuated WNV-NS5-E218A was constructed from WNV 3356 strain as described previously [43, 44] and was passaged once in Vero cells, as described previously [45]. Stock titers for all viruses were determined using BHK21 cells for viral plaque assay as previously described [46].

### Mouse experiments

All mice used in these experiments were male C57BL/6 inbred mice obtained commercially (The Jackson Laboratory). All mice were housed in pathogen-free facilities at the Washington University School of Medicine. PLX5622 was provided by Plexxikon Inc. and formulated in AIN-76A rodent diet at a dose of 1200 mg/kg by Research Diets. Mice were provided PLX5622 or control AIN-76A chow starting at 6 weeks of age for 2 weeks. For viral inoculations, mice were anesthesthetized with a cocktail of ketamine/xylazine/acepromazine, then inoculated subcutaneously via footpad injection (f.p., 50 μl) or intracranially (i.c., 10 μl) with a guided 29G needle into the brain’s third ventricle.

### Viral tissue burden and viremia quantification

For in vivo virus replication experiments, mice were infected with WNV and euthanized at specific days post-infection, as indicated. For tissue collection, mice were deeply anesthetized with ketamine/xylazine/acepromazine, blood collected in serum separator tubes, then transcardially perfused with sterile Dulbecco’s phosphate-buffered saline (dPBS; Gibco). The spleen and kidneys were collected, then the brain and spinal cord removed and microdissected. All organs were snap frozen, weighed, and then homogenized in dPBS. Virus was titered by standard plaque assay with BHK21 cells, as described previously [46]. Serum viremia was measured using TaqMan quantitative RT-PCR (qRT-PCR) primers and probe listed in Table 1, as described previously [47].

**Table 1:** Primer and probe sequences used for qRT-PCR

Target	Primer sequence (5′➔3′)
*GAPDH*	
Fwd	GGC AAA TTC AAC GGC ACA GT
Rev	AGA TGG TGA TGG GCT TCC C
*IFNα*	
Fwd	CTT CCA CAG GAT CAC TGT GTA CCT
Rev	TTC TGC TCT GAC CAC CTC CC
*IFNβ*	
Fwd	CTG GAG CAG CTG AAT GGA AAG
Rev	CTT CTC CGT CAT CTC CAT AGG G
*IFNγ*	
Fwd	AAC GCT ACA CAC TGC ATC TTG G
Rev	GCC GTG GCA GTA ACA GCC
*TNFα*	
Fwd	GCA CAG AAA GCA TGA TCC G
Rev	GCC CCC CAT CTT TTG GG
*iNOS*	
Fwd	GGA GCC TTT AGA CCT CAA CAG A
Rev	TGA ACG AGG AGG GTG GTG
*CD86*	
Fwd	ACG ATG GAC CCC AGA TGC ACC A
Rev	GCG TCT CCA CGG AAA CAG CA
*CD68*	
Fwd	CCA CAG GCA GCA CAG TGG ACA
Rev	TCC ACA GCA GAA GCT TTG GCC C
*Arg1*	
Fwd	TTA GGC CAA GGT GCT TGC TGC C
Rev	TAC CAT GGC CCT GAG GAG GTT C
*TGFβ*	
Fwd	TAC TAT GCT AAA GAG GTC ACC C
Rev	CTT CCC GAA TGT CTG ACG TAT TG
*IL10*	
Fwd	TTG GAA TTC CCT GGG TGA GAA
Rev	GGA GAA ATC GAT GAC AGC GC
*WNV*	
Fwd	TCA GCG ATC TCT CCA CCA AAG
Rev	GGG TCA GCA CGT TTG TCA TTG
Probe	/56-FAM/TGC CCG ACC /ZEN/ ATG GGA GAA GCT C/3IABkFQ/

### Leukocyte isolation and flow cytometry

For flow cytometry experiments, mice were deeply anesthetized with ketamine/xylazine/acepromazine, then blood was collected in 5 μM EDTA in dPBS (Gibco). Mice were transcardially perfused with dPBS, and then, the spleen, brain, popliteal LNs, and femur were removed. Bone marrow was collected from the femur by centrifuging the bone in a 0.65-ml microtube punctured with an 18G needle nested inside a 1.7-ml tube. Brain tissue was minced and digested in a HBSS (Gibco) containing 0.05% collagenase D (Sigma), 0.1 μg/ml TLCK trypsin inhibitor (Sigma), 10 μg/ml DNase I (Sigma), and 10 mM Hepes pH 7.4 (Gibco) for 1 h at 22 °C with shaking. Brain, spleen, and LN tissues were pushed through a 70-μm strainer and centrifuged at 500*g* for 10 min. Brain cell pellets were resuspended in 37% isotonic Percoll (GE healthcare) and centrifuged at 1200*g* for 30 min to remove myelin debris, and pellet was resuspended in dPBS. Red blood cells were lysed in blood, spleen, and bone marrow samples with ACK lysing buffer (Gibco) for 5 min, then centrifuged at 500*g* for 10 min and resuspended in dPBS. Prior to immunostaining, all cells were blocked with 1:50 TruStain FcX anti-mouse CD16/32 (Clone 93, Biolegend, Cat 101320) for 5 min. Cells were stained with antibodies, as indicated for 15 min at 22 °C, then washed thrice with dPBS, and fixed with 2% paraformaldehyde (PFA). Data were collected with a BD LSR Fortessa X-20 flow cytometer and analyzed with FlowJo software.

### Flow cytometry antibodies

All used at 1:200: CD11b (Clone M1/70, Biolegend, Cat 10137), CD45 (Clone 30-F11, eBioscience, Cat 56-0451-82), MHCII (Clone M5/114.15.2, Biolegend, Cat 107626), CD86 (Clone GL1, BD Biosciences, 553691), CD80 (Clone 16-10A1, Biolegend, 104706), CD11c (Clone N418, Biolegend, Cat 117335), CD103 (Clone 2E7, eBioscience, Cat 48-1031-80), CD8a (Clone 53-6.7, Biolegend, Cat 100712), CD4 (Clone RM4-5, BD Biosciences, Cat 550954), CD69 (Clone H1.2F3, eBiosciene, Cat 12-0691-81), Ly6C (Clone HK1.4, Biolegend, Cat 128003), Ly6G (Clone 1A8, Biolegend, Cat 127616), CD160 (Clone 7H1, Biolegend, Cat 143007), Rat-anti-P2RY12 (Clone S16007D, Biolegend, Cat 848002), and Goat-anti-Rat-AlexaFluor 350 (Invitrogen, Cat A21093). WNV-specific CD8+ T cells were identified with fluorescent-labeled immunodominant *D*^*b*^-restricted NS4B peptide.

### Quantitative RT-PCR

RNA was isolated from tissue homogenates using Qiagen RNeasy kit according to the manufacturer’s instructions. RNA was treated with DNAse, then cDNA was synthesized using random primers and MultiScribe reverse transcriptase (Applied Biosystems). A single reverse-transcriptase master mix was used to reverse-transcribe all samples in order to minimize differences in reverse-transcription efficiency using the following conditions: 25 °C for 10 min, 48 °C for 30 min, and 95 °C for 5 min. For all primers except WNV, qRT-PCR was performed using Power SYBR Green PCR mastermix, and data are reported as ΔCt (Ct_target_ − Ct_GAPDH_). Viral RNA was isolated from serum using Qiagen Viral RNA kit. A standard curve of viral genome with known PFU and serum samples were quantified using TaqMan reagents and the primers and probe listed in Table 1.

### Tunel stain, immunohistochemistry, and confocal microscopy

Following perfusion with ice cold PBS and 4% paraformaldehyde (PFA), brains were immersion-fixed overnight in 4% PFA, followed by cryoprotection in two exchanges of 30% sucrose for 48 h, then frozen in OCT (Fisher). Ten-micrometer sagittal sections were cut, then washed with PBS, and permeabilized with 0.1% Triton X-100/0.1% sodium citrate. Tunel reaction was prepared according to the manufacturer’s directions (Roche in situ cell death kit, TMR Red, Cat 12-156-792-910). Sections were immunostained for NeuN or Iba1 by blocking sections with 5% goat serum/0.1% Tween 20/PBS, then incubating overnight at 4 °C with rabbit-anti-NeuN (1:1000, Clone D3S3I, Cell Signaling Technology Cat 12943S) or rabbit-anti-Iba1 (1:1000, Wako Cat 019-19741). Sections were washed with 0.1% Tween 20/PBS, then incubated with goat-anti-rabbit-AF488 (4 μg/ml, Invitrogen, Cat A11008), counterstained with 1 μg/ml DAPI, and coverslipped with Prolong Gold Antifade Mountant (ThermoFisher, Cat P36930). Z-stack images (seven images separated by 1 μm) were captured using a Zeiss LSM 510 laser-scanning confocal microscope at × 40 objective magnification then compressed into a maximum intensity projection with accompanying software. For each region of each mouse, three to six images were taken from two different sagittal sections spaced at least 50 μm apart. The number of DAPI-positive nuclei also positive for NeuN and Tunel were counted by an individual blinded to the conditions.

### Statistical analysis

Statistical analyses were performed using Prism 7.0 (GraphPad Software). All data were analyzed using an unpaired *t* test or two-way ANOVA with Sidak’s post-test to correct for multiple comparisons, as indicated in the corresponding figure legends. A *P* value < 0.05 was considered significant.

## Results

### CSF1R antagonism depletes myeloid populations within peripheral blood and CNS

**Fig. 1:**
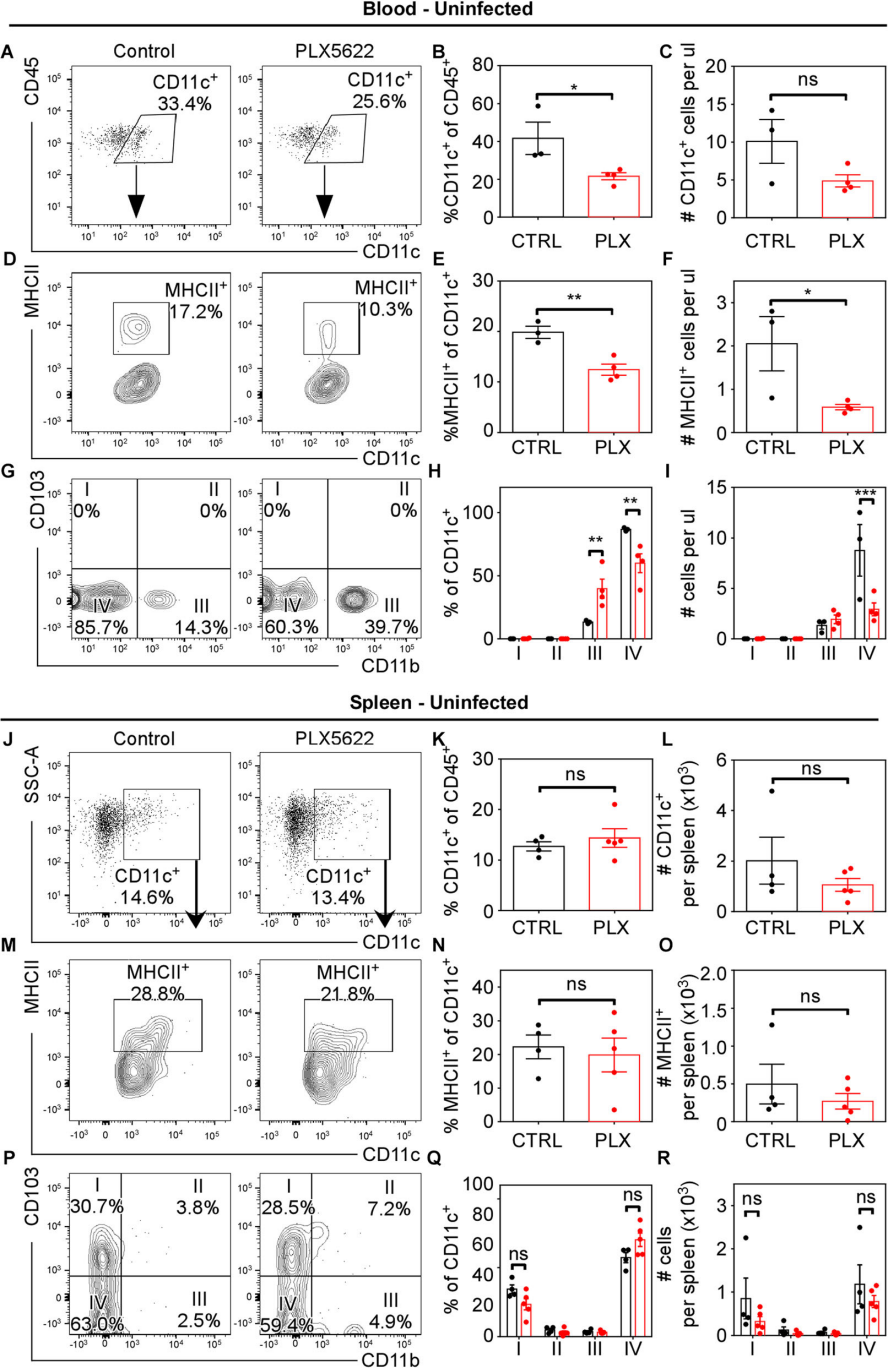
CSF1R antagonism reduces circulating APC populations in uninfected mice. Mice were fed PLX5622 chow or control chow for 2 weeks, and APCs were assessed in blood (**a**–**i**) and spleen (**j**–**r**). **a**, **j** Representative flow cytometry plots of CD11c expression on CD45^+^ cells. **b**, **k** Quantification of percentages and **c**, **l** total numbers of CD45^+^CD11c^+^ cells. **d**, **m** Representative flow cytometry plots of MHCII expression on CD11c^+^CD45^+^. **e**, **n** Quantification of percentages and **f**, **o** total numbers of MHCII^+^CD11c^+^CD45^+^ cells. **g**, **p** Representative flow cytometry plots of CD103 vs CD11b expression on CD11c^+^CD45^+^ cells. **h**, **q** Quantification of percentages and **i**, **r** total numbers of (I) CD103^+^CD11b^neg^CD11c^+^CD45^+^, (II) CD103^+^CD11b^+^CD11c^+^CD45^+^, (III) CD103^neg^CD11b^+^CD11c^+^CD45^+^, and (IV) CD103^neg^CD11b^neg^CD11c^+^CD45^+^ cells. For quantification panels, each symbol represents an individual control (black) or PLX5622-treated (red) mouse, and bars indicate mean ± SEM. Data shown represent analysis from one experiment with three to five mice per group, repeated in three independent experiments. Statistical significance was calculated using two-way ANOVA with Sidak’s multiple comparisons test. For all data: ns, not significant at *P* < 0.05; *P < 0.05; **P < 0.01; ***P < 0.001. CTRL: Control; PLX: PLX5622

CSF1R signaling is essential for the development of mononuclear phagocytes including microglia, but pharmacological antagonism has been reported to selectively deplete microglia [48]. To examine the impact of CSF1R antagonism on both peripheral and CNS mononuclear cells, we used PLX5622, which reportedly depletes microglia in as little as 3 days and may be sustained for at least 6 weeks [37, 41]. Mice were provided either control chow or PLX5622-embedded chow for 2 weeks, after which microglia numbers were assessed by flow cytometry using the microglia-specific marker P2RY12 (Additional file 1) and by immunohistochemistry using Iba1 (Additional file 2). Quantification shows that 2 weeks of PLX5622 treatment reduced microglia in the CNS by 90–95% (Additional file 1 A–F, P, Q). Specificity of P2RY12 for CNS-resident microglia is demonstrated by lack of staining in leukocytes isolated from peripheral immune compartments (Additional file 1 G–O). Within the CNS, PLX5622 treatment significantly depleted microglia across hippocampal, cortical, and cerebellar brain regions (Additional file 2). To determine whether PLX5622 treatment depleted mononuclear cells in peripheral immune compartments, we assessed numbers of antigen-presenting cells (APCs, Fig. 1) and T cells (Additional file 3) in the spleen and blood of uninfected mice in control versus PLX5622-treated mice. PLX5622 treatment significantly reduced circulating CD11c^+^ (Fig. 1a–c), MHCII^+^CD11c^+^ (Fig. 1d–f), and CD11b^neg^CD11c^+^ (Fig. 1g–i) cells in the blood. In the spleen, however, there was no significant difference in populations of APCs with PLX5622 treatment (Fig. 1j–r). In addition, no significant difference in populations of T cells was detected in uninfected mice in either compartment (Additional file 3). Because of the sensitivity of microglia and APCs to CSF1R antagonism, we assessed whether CSF1R antagonism depleted CD11b^+^, Ly6C^+^, and Ly6G^+^ cells in the blood, spleen, and bone marrow of PLX5622-treated versus control-treated uninfected mice. Flow cytometry analysis revealed no significant differences in populations of these cells in uninfected mice (Additional file 4). Together, these data indicate that circulating APCs, but not bone marrow or splenic mononuclear cells, are decreased in the context of CSF1R antagonism.

### CSF1R antagonism increases susceptibility of mice to fatal WNV infection

**Fig. 2:**
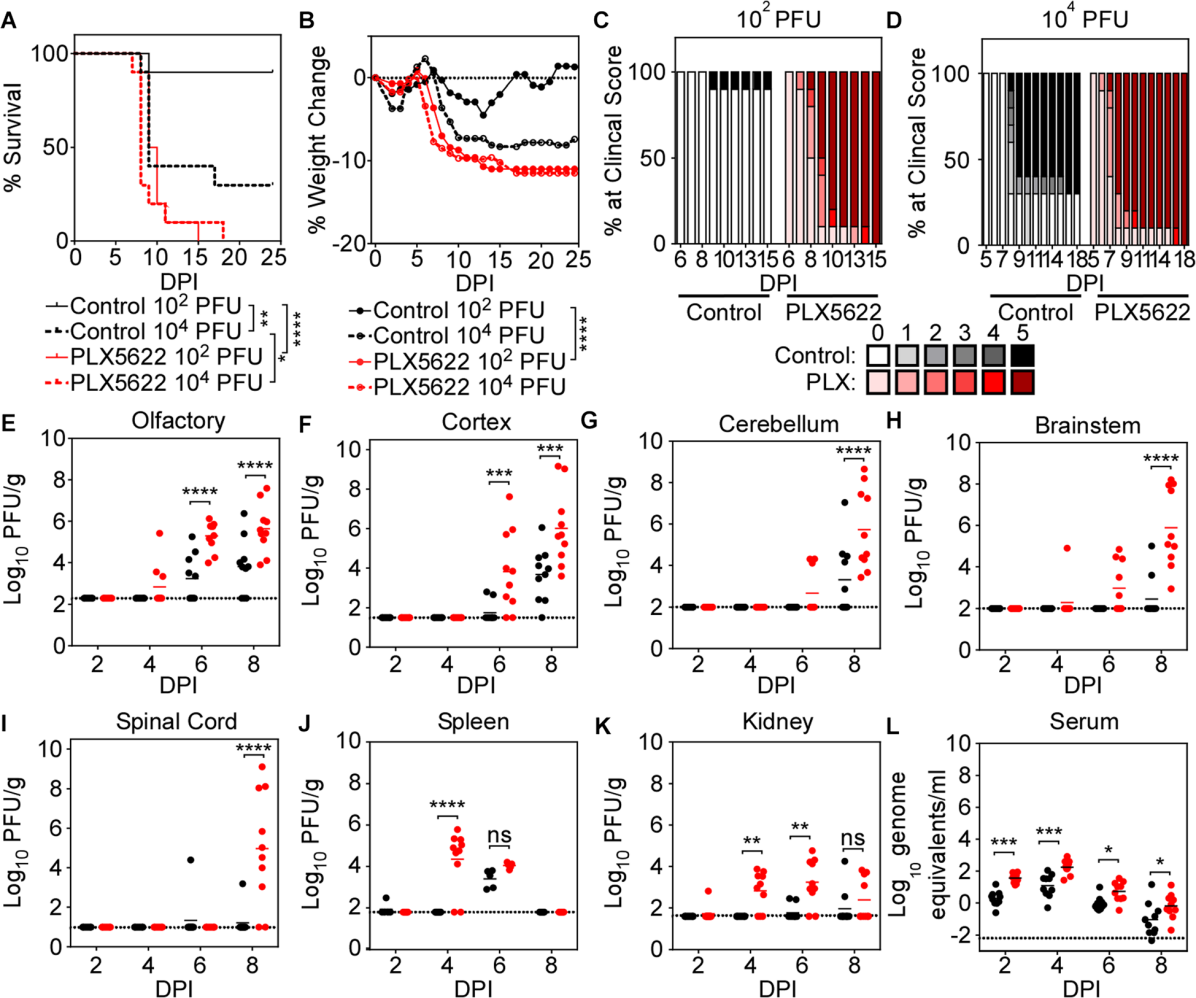
PLX5622-treated mice exhibit increased mortality, increased encephalitis score, and impaired virologic control compared with control mice following peripheral infection with WNV-NY. **a**–**d** Mice were fed chow containing PLX5622 or control chow for 2 weeks, then infected with 10^2^ or 10^4^ PFU via footpad infection, then monitored for (**a**) mortality, (**b**) weight loss, and (**c**, **d**) encephalitis score for up to 25 dpi. **a** Survival curves show a significant increase in mortality in mice treated with PLX5622 compared with control-treated mice infected with either viral inoculum dose, as calculated by log-rank (Mantel-Cox) test. **b** Weight was measured daily during acute illness as a measure of illness. After death, the last measured weight was carried through to the end of the experiment. Significantly greater weight loss was measured in PLX5622-treated mice compared with controls infected with 10^2^ PFU as calculated by two-way ANOVA with matched values comparing group means without multiple comparisons. **c** Clinical scores of PLX5622 or control-treated mice infected with 10^2^ PFU or **d** 10^4^ PFU as indicated during acute illness. 0 = subclinical, 1 = hunched/ruffled fur, 2 = altered gate/slow movement, 3 = no movement, but responsive to stimuli, 4 = moribund, 5 = dead. **e**–**k** Tissue viral loads as measured by plaque assay at 2, 4, 6, and 8 dpi following infection with 10^2^ PFU in control (black) or PLX5622-treated (red) mice. **l** Serum viral loads as measured by qRT-PCR. **e**–**l** Data are presented as scatter plots with each mouse represented by a dot with the SEM indicated by a line. Dotted lines in **e**–**l** indicate assay limit of detection. All data presented are the compilation of two independent experiments with 10 mice per group. Statistical significance was calculated using two-way ANOVA with Sidak’s multiple comparisons test. For all data: ns, not significant at *P* < 0.05; *P < 0.05; **P < 0.01; ****P < 0.0001

To determine whether CSF1R antagonism impacts survival from WNV infection, mice were fed PLX5622-embedded chow for 2 weeks, then infected with WNV-NY [42] at either 10^2^ or 10^4^ PFU via footpad (f.p.) inoculation. Consistent with published data [8, 49], 30% of control-treated mice survived infection with 10^4^ PFU and 90% of control-treated mice survived infection with 10^2^ PFU; however, mice treated with PLX5622 universally succumbed to infection with either inoculum titer (Fig. 2a). This was associated with significantly increased weight loss (Fig. 2b) and more severe clinical score (Fig. 2c, d) in PLX5622-treated compared with control-treated mice infected with 10^2^ PFU WNV-NY. To determine whether increased mortality caused by PLX5622 treatment is associated with increased viral replication in peripheral or CNS tissues, tissue viral loads were assessed by plaque assay, and serum viral loads were determined by quantitative RT-PCR (qRT-PCR) at 2, 4, 6, and 8 days post-infection (dpi). In the CNS, virus was first detected in the olfactory bulb (Fig. 2e), then sequentially in more caudal regions including the cortex (Fig. 2f), cerebellum (Fig. 2g), brainstem (Fig. 2h), and spinal cord (Fig. 2i). Compared with control-treated animals, PLX5622-treated mice exhibited higher viral titers in the olfactory bulb and cortex at 6 and 8 dpi and in the cerebellum, brainstem, and spinal cord at 8 dpi; however, viral titers were also significantly higher in peripheral tissues of PLX5622-treated mice including the spleen at 4 dpi (Fig. 2j), kidney at 4 and 6 dpi (Fig. 2k), and serum at 2, 4, 6, and 8 dpi (Fig. 2l). PLX5622 treatment did not cause increased BBB permeability in infected mice (Additional file 5). Once WNV-NY enters the CNS via intracranial (i.c.) inoculation, PLX5622 treatment did not affect neuronal permissivity to infection (Additional file 6). Together, these data indicate a loss of immune-mediated virologic control in both peripheral and CNS tissues in CSF1R antagonist-treated mice, consistent with prior data suggesting that CSF1R signaling plays important roles in the function of both peripheral and CNS myeloid cells [50–52].

**Fig. 3:**
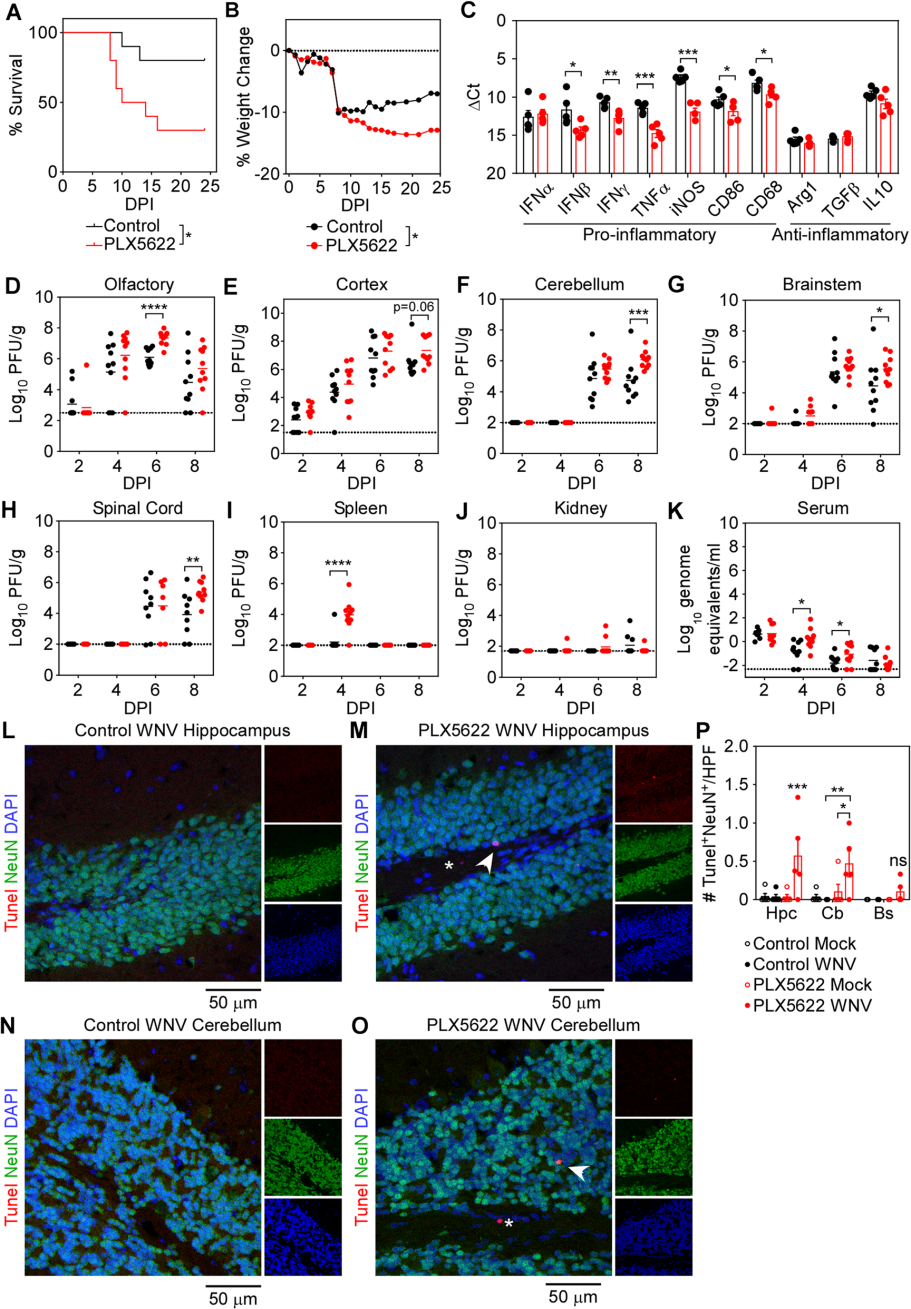
CSF1R antagonism increases mortality and CNS viral burdens during i.c. infection with WNV-NS5-E218A. Mice were fed chow containing PLX5622 or control chow for 2 weeks, infected i.c. with WNV-NS5-E218A at 10^4^ PFU, then monitored for **a** mortality and **b** weight loss for up to 25 dpi. **a** Survival curves show a significant increase in mortality in mice treated with PLX5622 compared with control as calculated by log-rank (Mantel-Cox) test. **b** Weight was measured daily during acute illness as a measure of illness. After death, the last measured weight was carried through to the end of the experiment. Significantly greater weight loss was measured in PLX5622-treated mice compared with controls as calculated by two-way ANOVA with matched values comparing group means without multiple comparisons. For **a** and **b**, data represent the compilation of two independent experiments with 10 mice per group. **c** At 8 dpi, cortical brain tissue was extracted and relative transcript levels in tissue homogenates were measured by SYBR qRT-PCR for indicated cytokines. Data for individual mice were normalized to *Gapdh.* Data are presented as mean ± SEM of three to five mice from one experiment. Statistical significance between treatment groups for each cytokine was calculated by *t* test. **d**–**j** Tissue viral loads were measured by plaque assay at 2, 4, 6, and 8 dpi in control (black) or PLX5622-treated (red) mice. **k** Serum viral loads as measured by qRT-PCR. **d**–**k** Data are presented as scatter plots with each mouse represented by a dot with the mean indicated by a line. For **d**–**k**, data represent the compilation of two-independent experiments with 10 mice per group. Dotted lines in **d**–**k** indicate assay limit of detection. **l**–**o** Representative confocal microscopic images of Tunel (red), NeuN (green), and DAPI (blue) of hippocampus (**l**, **m**) and cerebellum (**n**, **o**) of control- (**l**, **n**) or PLX5622-treated (**m**, **o**) mice infected i.c. with WNV-NS5-E218A at 6 dpi. Arrow heads point to DAPI-positive nuclei positive for both Tunel and NeuN. Asterisks denote DAPI- and Tunel-positive but NeuN-negative nuclei. **p** Quantification of confocal microscopic images for number of DAPI-positive nuclei positive for Tunel and NeuN in the hippocampus (Hpc), cerebellum (Cb), and brainstem (Bs). The number of Tunel+ NeuN+ nuclei per high-power field (HPF) was quantified from three to six images captured at × 40 per brain region across two separate brain sections for each of five independent mice collected in one experiment. Each symbol represents the average number for an individual mouse, with bar indicating mean ± SEM. Statistical significance was calculated using two-way ANOVA with Sidak’s multiple comparisons test. For all data: ns, not significant at *P* < 0.05; *P < 0.05; **P < 0.01; ****P < 0.0001

To determine whether microglia depletion impacts innate immune mechanisms of virologic control specifically within the CNS, mice were fed control or PLX5622-embedded chow for 2 weeks, then i.c. infected with an attenuated strain of WNV (WNV-NS5-E218A) at 10^4^ PFU. The NS5-E218A point mutation abolishes 2′-*O*-methyltransferase activity, which functions to methylate the 5′ cap of viral RNA and facilitate escape of IFN-induced proteins with tetratricopeptide repeats (IFIT)-mediated suppression; thus, this attenuation limits viral replication by increasing sensitivity of the virus to type I IFN-mediated innate immune mechanisms [45, 49]. Despite this attenuation, mice treated with PLX5622 were significantly more susceptible to lethal WNV-NS5-E218A infection than those receiving control chow (Fig. 3a). Weight loss was also significantly increased in PLX5622-treated mice (Fig. 3b). Consistent with this, viral titers measured by plaque assay at 2, 4, 6, and 8 dpi in the olfactory bulb (Fig. 3d), cortex (Fig. 3e), cerebellum (Fig. 3f), brainstem (Fig. 3g), and spinal cord (Fig. 3h) were significantly higher in PLX5622-treated compared in control-treated mice infected with WNV-NS5-E218A. As virus was inoculated into the cortex, it was detected there at 2 dpi, followed by spread to the olfactory bulb by 4 dpi, and cerebellum, brainstem, and spinal cord by 6 dpi. PLX5622 treatment also led to decreased proinflammatory cytokine expression in the cortex at 8 dpi (Fig. 3c) and increased neuronal apoptosis in the hippocampus and cerebellum at 6 dpi (Fig. 3l–p) after i.c. infection with WNV-NS5-E218A.

As expected, WNV-NS5-E218A was barely detected in the kidney (Fig. 3j), and the virus was steadily cleared from the serum (Fig. 3k) in both control- and PLX5622-treated mice. However, significantly higher levels of virus were detected in the spleen of PLX5622- versus control-treated, WNV-NS5-E218A-infected mice at 4 dpi (Fig. 3i), which was consistent with data obtained during f.p. infection with WNV-NY. Overall, CSF1R antagonism leads to enhanced mortality and loss of virologic control in both the periphery and CNS regardless of the WNV strain utilized, suggesting a critical role for this molecule in antiviral immunity.

### CSF1R signaling is required for activation of APC co-stimulatory function

**Fig. 4:**
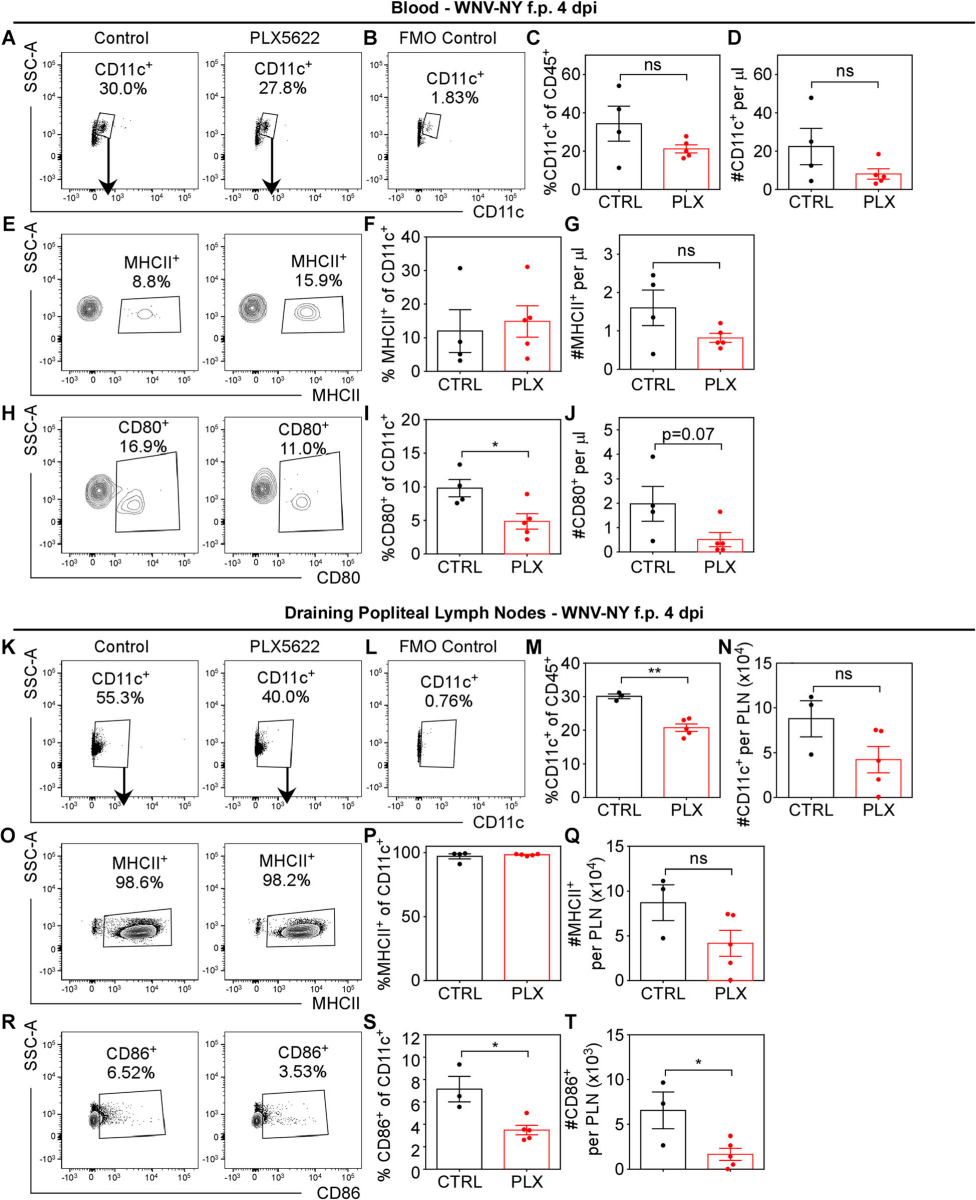
CSF1R antagonism reduces response of APCs in peripheral immune compartments following peripheral infection with WNV-NY. Mice were fed PLX5622 or control chow for 2 weeks, then infected via f.p. with WNV-NY (100 PFU). At 4 dpi, leukocytes were isolated from blood (**a**–**j**) and draining popliteal LNs (k–t). **a**, **k** Representative flow cytometry plots of CD11c expression on CD45^+^-gated cells and **b**, **l** fluorescence minus one (FMO) gating controls. **c**, **m** Quantification of percentages and **d**, **n** total numbers of CD11c^+^ CD45^+^ cells. **e**, **o** Representative flow cytometry plots of MHCII^+^ expression on CD11c^+^CD45^+^ cells. **f**, **p** Quantification of percentages and **g**, **q** total numbers of MHCII^+^CD11c^+^CD45^+^ cells. **h** Representative flow cytometry plots of CD86^+^ expression on CD11c^+^CD45^+^ cells. **i** Quantification of percentages and **j** total numbers of CD86^+^CD11c^+^CD45^+^ cells. **r** Representative flow cytometry plots of CD80^+^ expression on CD11c^+^CD45^+^ cells. **s** Quantification of percentages and **t** total numbers of CD80^+^CD11c^+^CD45^+^ cells. For quantification panels, each symbol represents an individual control- (black) or PLX5622-treated (red) mouse, and bars indicate mean ± SEM. Data shown represent analysis from one experiment with three to five mice per group. Statistical significance was calculated using unpaired *t* test in all panels except **d** and **n**, which used two-way ANOVA with Sidak’s multiple comparisons test. For all data: ns, not significant at *P* < 0.05; *P < 0.05; **P < 0.01

To determine whether alterations in APC populations impact antiviral immune responses, control- and PLX5622-treated mice were infected via f.p. with WNV-NY (100 PFU). After f.p. infection, WNV-infected cells traffic to draining popliteal lymph nodes (pLN) and stimulate an antiviral immune response [53]. At 4 dpi, a time-point of high serum viral loads (Fig. 2l), flow cytometric assessment revealed a trend towards decreased CD11c^+^CD45^+^ cells in the blood (Fig. 4a–d) and significantly reduced percentages and total numbers of CD11c^+^CD45^+^ cells in the pLN of PLX5622-treated compared with control-treated mice (Fig. 4k–n). There was no difference in numbers or percentages of MHCII^+^CD11c^+^CD45^+^ cells in either blood or pLN (Fig. 4e–g, o–q); however, the percentages of CD11c^+^CD45^+^ cells in the blood positive for co-stimulatory molecule CD80^+^ was significantly decreased in PLX5622- versus control-treated mice (Fig. 4h–j). In the pLN, the numbers and percentages of CD11c^+^CD45^+^ cells positive for co-stimulatory molecule CD86 were significantly reduced in PLX5622-treated mice compared with control mice (Fig. 4r–t). The impact of CSF1R antagonism on CD80 and CD86 expression appears to be tissue-dependent as the blood showed no difference in APCs expressing CD86 and the pLN showed no difference in APCs expressing CD80 (Additional file 7). Together, these data indicate that CSF1R antagonism decreases the expression of co-stimulatory B7 molecules on peripheral APC populations.

**Fig. 5:**
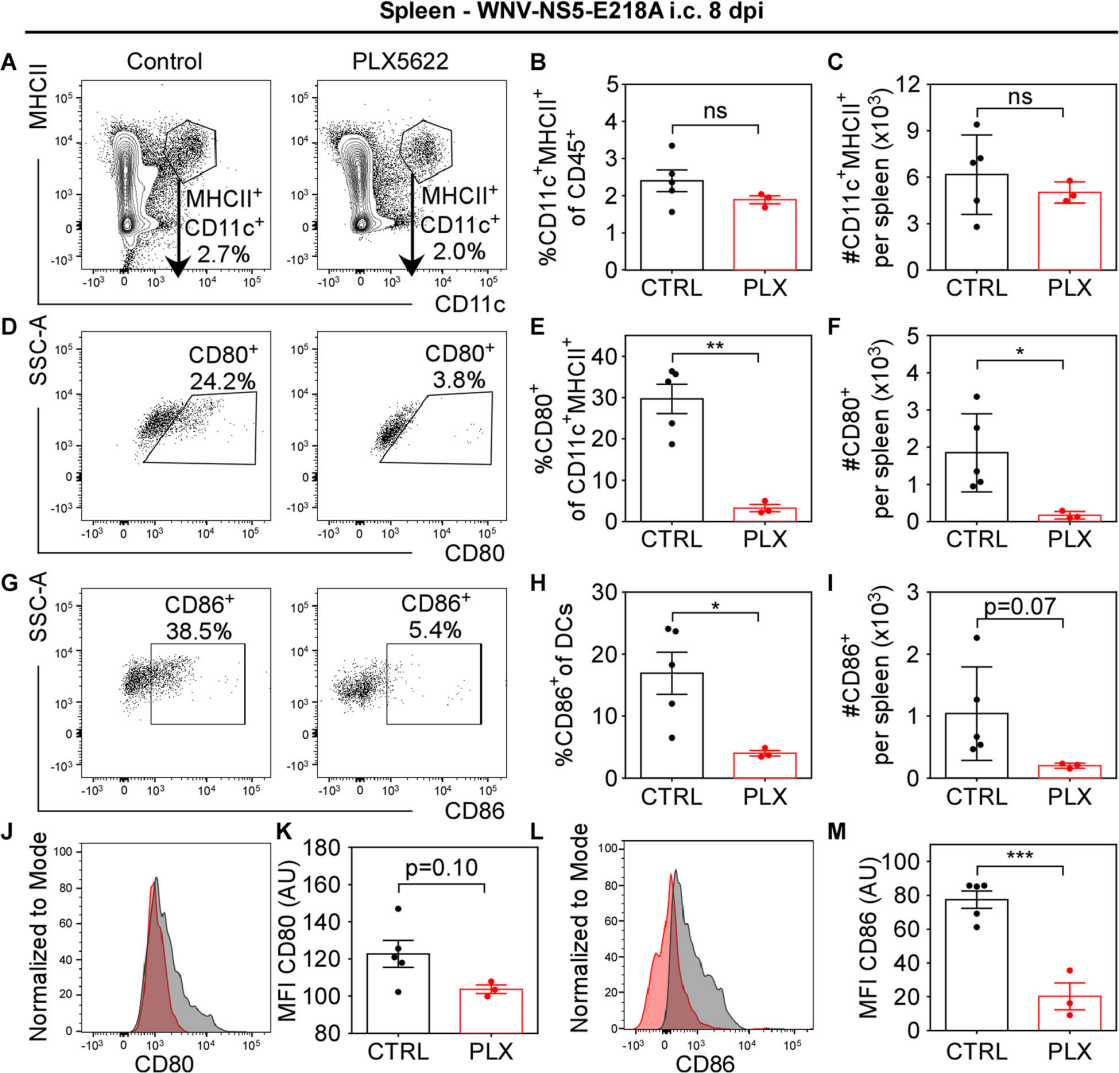
CSF1R antagonism reduces co-stimulatory signal 2 on splenic APCs during i.c. infection with WNV-NS5-E218A. Mice were fed PLX5622 chow or control chow for 2 weeks, then infected i.c. with WNV-NS5-E218A (10^4^ PFU). At 8 dpi, splenic leukocytes were analyzed. **a** Representative flow cytometry contour plots of CD11c and MHCII expression on CD45^+^-gated cells and **b** quantification of percentages and **c** total numbers of CD11c^+^MHCII^+^CD45^+^ cells. **d** Representative flow cytometry plots of CD80 expression on CD11c^+^MHCII^+^CD45^+^ cells. **e** Quantification of percentages and **f** total numbers of CD80^+^CD11c^+^MHCII^+^CD45^+^ cells. **g** Representative flow cytometry plots of CD86 expression on CD11c^+^MHCII^+^CD45^+^ cells. **h** Quantification of percentages and **i** total numbers of CD86^+^CD11c^+^MHCII^+^CD45^+^ cells. **j** Representative flow cytometry histograms of CD80 expression on CD11c^+^MHCII^+^CD45^+^ and **k** quantification of MFI. **l** Representative flow cytometry histograms of CD86 expression on CD11c^+^MHCII^+^CD45^+^ and **m** quantification of MFI. AU, arbitrary units. For quantification panels, each symbol represents an individual control- (black) or PLX5622-treated (red) mouse, and bars indicate mean ± SEM. Data represent analysis from one experiment with three to four mice per group. Statistical significance was calculated using unpaired two-way *t* tests. For all data: ns, not significant at *P* < 0.05; *P < 0.05; **P < 0.01

Because infectious virus was detected in the spleens of mice infected i.c. with WNV-NS5-E218A (Fig. 3i), splenic APCs were analyzed using flow cytometry at 8 dpi. Results indicated no significant difference in the numbers or percentages of MHCII^+^CD11c^+^CD45^+^ cells in the spleens of PLX5622-treated mice compared with control-treated mice (Fig. 5a–c). However, splenic APCs collected from PLX5622-treated mice exhibited significantly reduced populations of cells expressing co-stimulatory molecules CD80 (Fig. 5d–f) and CD86 (Fig. 5g–i) compared with control-treated mice. Furthermore, splenic MHCII^+^CD11c^+^CD45^+^ cells showed significant reduction in the expression level of CD86 (Fig. 5l, m) and non-significant reduction of CD80 (Fig. 5j, k) in PLX5622-treated compared with control-treated mice. This correlated with a small, but significant increase in the numbers of CD4^+^ and CD8^+^ T cells, but no difference in numbers or percentages of WNV-specific NS4B tetramer staining or CD69 positivity in spleens of PLX5622-treated mice compared with control-treated mice infected i.c. with WNV-NS5-E218A (Additional file 8). Together, these data indicate that CSF1R antagonism reduces the activation of inflammatory responses in peripheral APCs even without significant depletion.

### Loss of cellular sources of co-stimulatory signals in the CNS results in decreased T cell reactivation

**Fig. 6:**
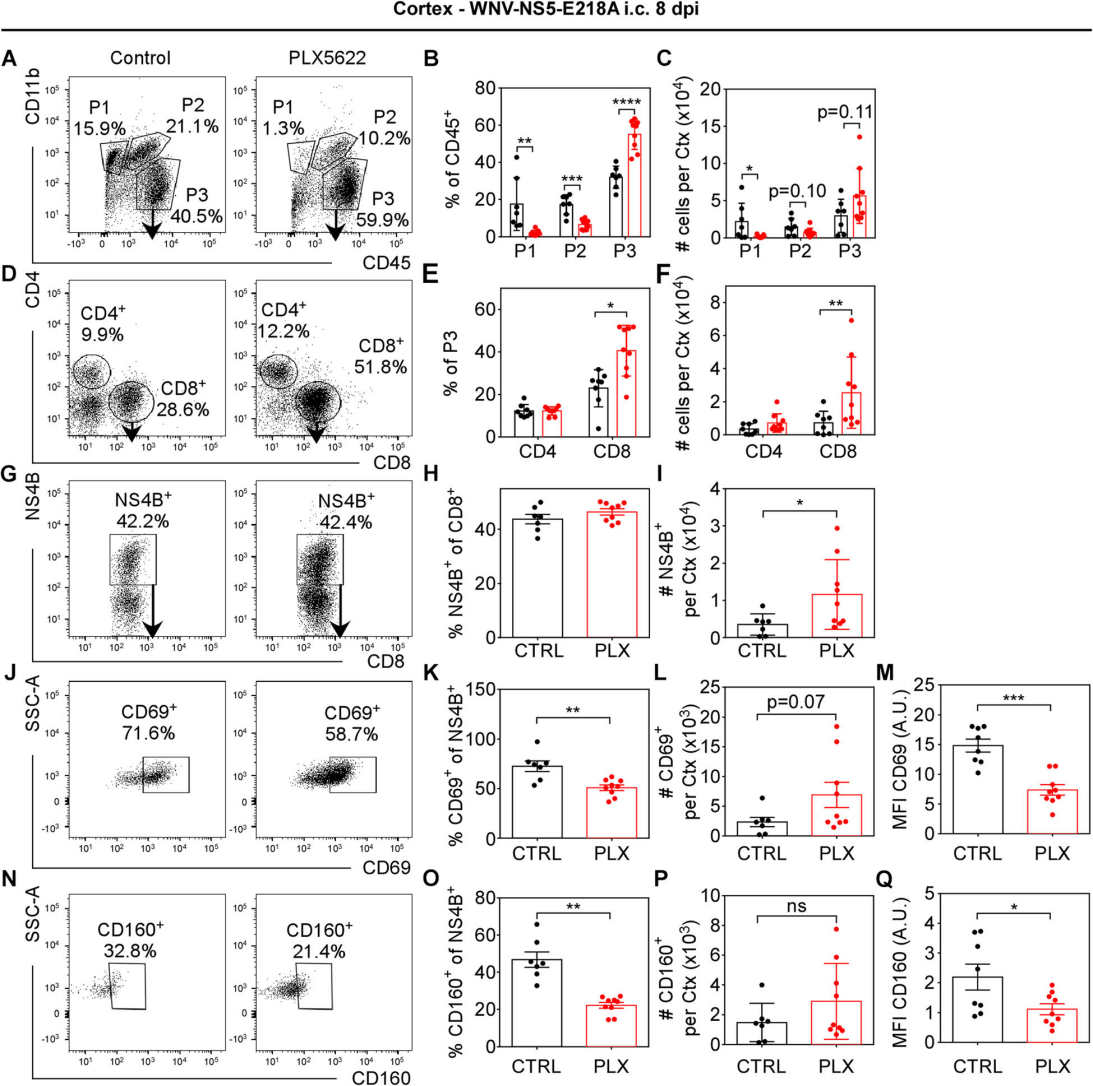
CSF1R antagonism does not hinder antiviral T cell recruitment to the CNS, but recruited T cells lack full activation during i.c. infection with WNV-NS5-E218A. Mice were fed PLX5622 chow or control chow for 2 weeks, then infected i.c. with WNV-NS5-E218A (10^4^ PFU). At 8 dpi, leukocytes were isolated from the cortex and analyzed by flow cytometry. **a** Representative flow cytometry plots of CD45^+^-gated cells analyzed by CD11b vs CD45 expression, identifying three populations of cells: CD11b^+^CD45^lo^ (P1), CD11b^+^CD45^hi^ (P2), and CD11b^neg^CD45^hi^ (P3). **b** Quantification of percentages and **c** number of cells in each gate. **d** Representative flow cytometry plots of CD4 vs CD8 expression and **e** quantification of percentages and **f** total numbers of CD4^+^ vs CD8^+^ cells within P3 gate. **g** Representative flow cytometry plots of NS4B^+^WNV-specific tetramer staining and **h** quantification of percentages and **i** total number of NS4B^+^CD8^+^CD45^+^ cells. **j** Representative flow cytometry plots of CD69 expression on NS4B^+^CD8^+^CD45^+^ cells and **k** quantification of percentages, **l** total numbers, and **m** MFI of CD69^+^NS4B^+^CD8^+^CD45^+^ cells. **n** Representative flow cytometry plots of CD160 expression on NS4B^+^CD8^+^CD45^+^ cells and **o** quantification of percentages, **p** total numbers, and **q** MFI of CD160^+^NS4B^+^CD8^+^CD45^+^ cells. MFI quantifications are shown as arbitrary units (AU) by normalizing each sample to the MFI of FMO negative control value [77]. For quantification panels, each symbol represents an individual control- (black) or PLX5622-treated (red) mouse, and bars indicate mean ± SEM. Data presented are the compilation of two independent experiments with seven to nine mice per group. Statistical significance was calculated using unpaired *t* tests with Welch’s correction. For all data: ns, not significant at *P* < 0.05; *P < 0.05; **P < 0.01; ***P < 0.001; ****P < 0.0001

Microglia are hypothesized to act as immediate responders to CNS pathogens as resident APCs that coordinate intra-parenchymal adaptive immune response [54]. Previous studies indicated that clearance of WNV in the CNS requires antiviral CD8^+^ T cells [19, 32, 55], and that microglia, astrocytes, and neurons express chemokines that attract mononuclear cells to the CNS [21, 56, 57]. To determine the impact of CSF1R antagonism on recruitment of these cells to the CNS, leukocytes isolated from the cortices of WNV-NS5-E218A-infected mice were assessed by flow cytometry at 8 dpi. As expected, CD45^lo^CD11b^+^ (P1) population, which is primarily microglia [58], remained significantly depleted in the PLX5622-treated compared with control-treated mice (Fig. 6a–c). The population of CD45^hi^CD11b^+^ (P2), which is primarily infiltrating monocytes/macrophages [58], were non-significantly reduced in the PLX5622-treated compared with control-treated mice. However, the number of CD45^hi^CD11b^neg^ (P3) lymphocyte population [58] were non-significantly elevated in PLX5622-treated compared with control mice, though the percentage of this population was significantly elevated (Fig. 6a–c). This increase was largely due to elevated numbers of CD8^+^ T cell infiltration in PLX5622-treated mice (Fig. 6d–f). Though there was no significant difference in the percentage of CD8^+^ T cells specific for the WNV immunodominant peptide between PLX5622 and control-treated mice, there were significantly more WNV-specific CD8^+^ T cells isolated from PLX5622 compared with control-treated cortex (Fig. 6g–i). Despite the increased infiltration of WNV-specific CD8^+^ T cells in PLX5622-treated mice, the percentages of cells expressing early activation marker CD69 and their level of expression were significantly reduced in PLX5622-treated compared with control-treated mice (Fig. 6j–m). Additionally, the percentage of WNV-specific CD8^+^ T cells positive for CD160 as well as the level of CD160 expression, which is specifically expressed on highly activated CD8^+^ T cells [59], were reduced in PLX5622-treated compared with control-treated mice. However, the numbers of NS4B+CD8+ cells positive for either CD69 or CD160 were non-significantly elevated in PLX5622-treated compared with control-treated cortices (Fig. 6l, p) due to the increased numbers of NS4B+CD8+ cells.

**Fig. 7:**
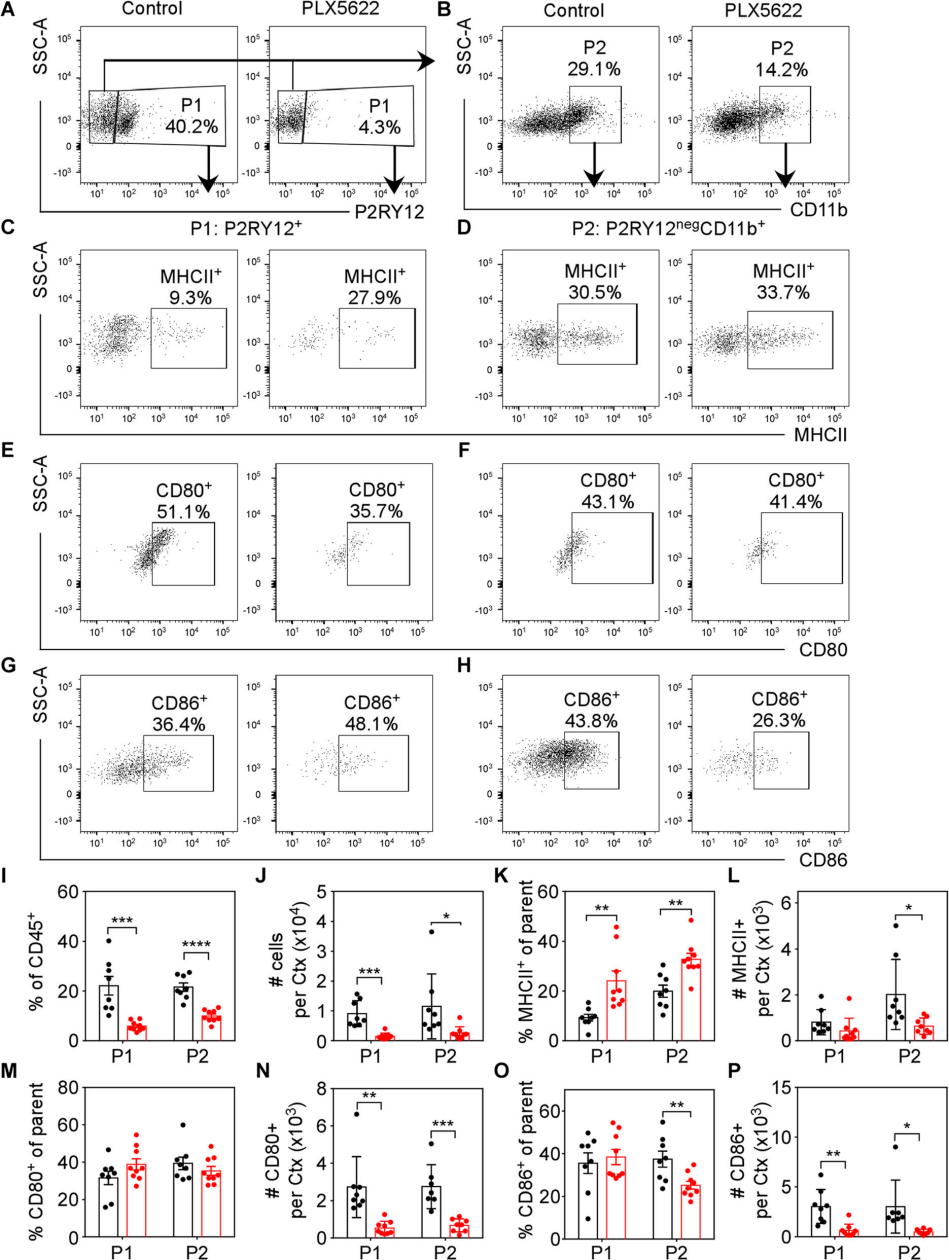
Microglia supply co-stimulatory signal 2 for T cell activation in the WNV-NS5-E218A-infected CNS. Mice were fed PLX5622 chow or control chow for 2 weeks, then i.c. infected with WNV-NS5-E218A (10^4^ PFU). At 8 dpi, leukocytes were analyzed from the cortex. **a** Representative flow cytometry contour plots of P2RY12 expression on CD45^+^-gated cells to identify resident microglia (P1). **b** Representative flow cytometry plots of CD11b expression on P2RY12^neg^CD45^+^ cells to identify infiltrating macrophages (P2). **c**, **d** Representative flow cytometry plots of MHCII expression on **c** P1 microglia and **d** P2 macrophages. **e**, **f** Representative flow cytometry plots of CD80 expression on **e** P1 microglia and **f** P2 macrophages. **g**, **h** Representative flow cytometry dot plots of CD86 expression on **g** P1 microglia and **h** P2 macrophages. **i**, **j** Quantification of flow cytometry analysis of **i** percentages and **j** total numbers of P1- vs P2-gated cells. **k** Quantification of percentages and **l** total numbers of MHCII^+^CD45^+^ cells within each P1 and P2 population. **m** Quantification of percentages and **n** total number of CD80^+^CD45^+^ cells within each P1 and P2 population. **o** Quantification of percentages and **p** total numbers of CD86^+^CD45^+^ cells within each P1 and P2 population. For quantification panels, each symbol represents an individual control- (black) or PLX5622-treated (red) mouse, and bars indicate mean ± SEM. Data presented are the compilation of two independent experiments with seven to nine mice per group. Statistical significance was calculated using multiple *t* tests with Welch’s correction. For all data: ns, not significant at *P* < 0.05; *P < 0.05; **P < 0.01; ***P < 0.001; ****P < 0.0001

Because numbers of infiltrating WNV-specific CD8^+^ T cells derived from the CNS of PLX5622-treated animals were increased, we hypothesized that their lack of local reactivation may be the result of loss of co-stimulatory signals and/or inflammatory cytokines (Fig. 3c) in the CNS. In order to distinguish resident microglia from infiltrating monocytes/macrophages, CNS myeloid cells were examined for expression of the microglia-specific marker P2RY12 (Fig. 7a). Infiltrating monocytes/macrophages were then identified via gating of P2RY12^neg^CD11b^+^ cells (Fig. 7b). Populations of both CNS-derived P2RY12^+^CD45^+^ microglia (P1, Fig. 7a) and CD11b^+^P2RY12^neg^CD45^+^ monocytes/macrophages (P2, Fig. 7b) were reduced in PLX5622-treated compared with control-treated mice (Fig. 7i, j). While no differences in the overall number of cells expressing MHCII were observed in P2RY12^+^CD45^+^, a significant reduction of MHCII^+^CD11b^+^P2RY12^neg^CD45^+^ was seen (Fig. 7c, d, l). This corresponded with a relative increase in the percentage of MHCII^+^ cells in both P2RY12^+^CD45^+^ and CD11b^+^P2RY12^neg^CD45^+^ populations due to the decreased numbers of total P2RY12^+^CD45^+^ and CD11b^+^P2RY12^neg^CD45^+^ cells in the cortices of PLX5622-treated mice compared with control-treated mice (Fig. 7k). However, the numbers of P2RY12^+^CD45^+^ cells expressing B7 co-stimulatory signals CD80 and CD86 were significantly reduced in the CNS of PLX5622-treated mice compared with control-treated animals (Fig. 7e–h, m–p). The percentage of remaining P2RY12^+^CD45^+^ cells expressing CD80 or CD86 were not reduced (Fig. 7m, o); however, the percentage of remaining CD11b^+^P2RY12^neg^CD45^+^ cells expressing CD86 was significantly reduced (Fig. 7o), similar to results seen in peripheral APCs (Figs. 4, 5) Together, these data suggest that resident microglia and infiltrating macrophages are important sources of CD80 and CD86 in the CNS, and PLX5622 treatment depletes these cellular sources of co-stimulatory molecules.

## Discussion

Recent studies have reported increased viral titers in the CNS of mice treated with CSF1R antagonists [36, 60, 61]; however, the mechanisms underlying this loss of virologic control and the effects on peripheral immune responses have not been well characterized. We sought to determine the role of CSF1R signaling in viral clearance of both systemic and neurotropic infection. Our results indicate that antagonism of CSF1R signaling limits virologic control within the CNS via reduction of critical APCs that express co-stimulatory B7 molecules, CD80 and CD86, necessary for local reactivation of antiviral CD8^+^ T cells. Our data also indicate that CSF1R antagonism reduces expression of co-stimulatory B7 molecules on peripheral APCs, contributing to loss of virologic control in the periphery and dissemination of virus to the CNS. As previously reported, WNV-NY inoculated into mice via f.p. is detected first within the olfactory bulb then spreads caudally through the cerebral cortex to the brain stem with significant virus not detected in the cerebellum until 8 dpi [20]. CSF1R antagonism with PLX5622 leads to increased viral replication within each of these CNS regions and also in the peripheral organs: the spleen, kidney, and serum. In contrast to CNS titers, the virus is cleared from the spleen and serum by 8 dpi in WNV-infected, control, and PLX5622-treated mice. Notably, we detected expansion of viral tropism with significant viral replication in the kidney of PLX5622-treated mice at 4 and 6 dpi compared with control-treated animals. These data suggest that renal macrophages may be sensitive to CSF1R antagonism, which is consistent with a study utilizing a model of recovery from acute kidney injury in which CSF1R inhibition with GW2580 reportedly decreased both kidney macrophage proliferation and their polarization towards wound-healing phenotypes [62]. In order to better isolate the role of microglia during neurotropic infection, we examined the effect of PLX5622 in an established murine model of CNS infection using an attenuated strain of WNV (WNV-NS5-E218A), which lacks 2′-*O*-methyltransferase activity, thus increasing sensitivity to IFIT-mediated suppression and limiting virulence in peripheral organs with intact immunity [8, 45, 49]. Mice infected i.c. with WNV-NS5-E218A exhibited increased viral titers in the CNS and increased lethality with PLX5622 treatment compared with control treatment. These data suggest that loss of CNS immunity increases lethality of WNV infection.

Clearance of WNV within the CNS requires local reactivation of antiviral CD8^+^ T cells for efficient T cell-mediated adaptive immune responses [55, 63]. Under homeostatic conditions, microglia express very low levels of co-stimulatory B7 molecules (CD80/CD86); however, microglia upregulate expression of these molecules in response to inflammatory cytokines [54, 64]. Our results show PLX5622 treatment decreased expression of these molecules by depleting their cellular sources in the CNS, which include both resident microglia and infiltrating monocytes/macrophages. In the periphery, PLX5622 also reduced cellular expression of B7 molecules on splenic, blood, and pLN APCs. Under certain conditions, other co-stimulatory molecules are able to compensate for the loss of B7 signals in T cell reactivation. For example, expansion of lymphocytic choriomeningitis virus-specific antiviral T cells can be driven by either alternative co-stimulatory TNFR superfamily members or by enhanced expression of antiviral cytokines, such as high levels of type I IFNs [65]. However, during WNV encephalitis, PLX5622 treatment also significantly decreased transcriptional expression of both TNF and IFNβ. Together, our results indicate that CSF1R antagonism contributes impaired local reactivation of antiviral T cells within the CNS by depleting co-stimulatory signals including both B7 molecules and inflammatory cytokines.

In addition to augmenting antiviral T cell activation, many cytokines have essential protective roles during WNV infection. Infected human monocyte-derived macrophages release IL-8, IFNα, IFNβ, and TNF [66, 67]. Microglia become activated in response to neurotropic WNV infection and upregulate expression of CXCL10, CXCL1, CCL5, CCL3, CCL2, TNF, and IL-6 [18]. Neurons and astrocytes in WNV-infected brains also release proinflammatory mediators including CCL2, CCL5, CXCL10, IL1β, IL-6, IL-8, and TNF, which promote lymphocyte trafficking to the CNS [19–21]. In fact, results from a recent study demonstrated that peripheral infection with WNV increased CNS expression of chemokines important for lymphocyte trafficking, including CCL2, CCL7, CXCL9, and CXCL10, in PLX5622-treated mice compared with control-treated mice [61]. Type I IFN response is widely accepted as the most immediate antiviral host response, essential for controlling viral replication during the initial infection. Mice lacking type I IFN signaling are highly vulnerable to uncontrolled WNV replication, exhibiting 100% mortality [68]. Clearance of viral infections, however, requires additional immune response including type II IFN, i.e., IFNγ, which is produced by activated CD8^+^ T cells. Mice deficient in IFNγ show higher peripheral viral load, increased CNS infection, and increased lethality [69]. Data described here show significantly decreased cytokine expression in PLX5622-treated mice, which may also contribute to the loss of virologic control via the above-described mechanisms.

The exact role of microglia during viral infection has been difficult to define because infiltrating monocytes can mask the impact of resident microglia. In a previous report, *IL34*^*−/−*^ mice, which have significantly reduced development of microglia, are more susceptible to lethal infection after WNV-NS5-E218A i.c. inoculation [36]. Similar to results reported here, *IL34*^*−/−*^ mice exhibited increased neuronal death compared with wildtype controls. In contrast with PLX5622-treated mice, *IL34*^*−/−*^ mice exhibited similar levels of viral burden compared with wildtype controls in the brain at 3 and 6 dpi. Numbers of infiltrating monocytes/macrophages and WNV-specific T cells were also similar between *IL34*^*−/−*^ and wildtype controls; however, the authors did not investigate antiviral T cell activation in this model [36]. In another model of flavivirus encephalitis, in which suckling mice were infected with dengue virus, depletion of microglia with liposome-encapsulated clodronate resulted in increased viral replication and reduced infiltration of IFNγ^+^CD8^+^ T cells [70]. Here, we report no reduction in CD8^+^ T cell infiltration; however, this difference may be due to the age of mice, virus, and/or method of microglial depletion. Another recent study reported use of PLX5622 in a model of neuroattenuated murine corona virus, mouse hepatitis virus (MHV). Similar to our results, PLX5622 treatment resulted in increased viral replication and lethality. In contrast to our results, PLX5622 treatment reduced expression of MHCII on microglia and macrophages and reduced CD4^+^ T cell response [60]. While CD4^+^ T cells can improve CD8^+^ T cell response, the dependence of CD8^+^ T cells on CD4^+^ T cells varies by virus, antigen exposure, tissue environment, and effector sites [71]. In models of MHV encephalitis, control of viral replication requires a collaborative effort between CD4^+^ and CD8^+^ T cells [71]. However, when virus replication induces activation of APCs, activation of CD8^+^ T cells is relatively CD4^+^ T cell-independent [71].

Recently, chronic activation of innate immune response by microglia is believed to be a major contributor to neurodegenerative conditions [72]. Genetic studies have confirmed the relevance of inflammatory response in common neurodegenerative diseases, such as Alzheimer’s disease and multiple sclerosis, indicating that neuroinflammation is likely implicated in the primary pathogenesis, rather than a secondary response [73]. We and others have reported that microglia impact neurocognitive function by executing complement-mediated synapse elimination [8, 74, 75]. Because of the neurological consequences of microglial activation, researchers have attempted to use CSF1R antagonism to deplete microglia in a variety of models to improve neurological function, including traumatic brain injury [38, 39], brain irradiation [50], myelin-induced catatonia [76], experimental autoimmune encephalomyelitis [40], and Alzheimer’s disease [41]. However, our results indicate that CSF1R antagonism may ultimately be detrimental to brain health due to loss of innate and adaptive immune response in the CNS, as well as decreased peripheral APC activation. Thus, in assessing the benefit of CSF1R antagonism, it will be important to consider the diminished immune response in both the CNS and peripheral immune compartments.

## Conclusions

This study provides evidence that CSF1R signaling is critically important for cellular immunity during WNV neurotropic infection. Pharmacologic antagonism of CSF1R signaling limits expression of co-stimulatory signals through depletion of CNS cellular sources and decreased expression on peripheral APCS. This reduces local restimulation of antiviral CD8^+^ T cells within the CNS and results in loss of virologic control and increased mortality. These data raise important considerations regarding the use of CSF1R antagonists for the treatment of neurological disorders associated with neuroinflammation.

## Additional files

Additional file 1: Example gating strategies and P2RY12 staining specificity. Mice were fed chow containing PLX5622 or control chow for 2 weeks, then P2RY12 microglia staining was assessed by flow cytometry in the (A–C) forebrain, (D–F) hindbrain, (G–I) blood, (J–L) spleen, and (M–O) bone marrow of uninfected mice. (A, D, G, J, M) Representative flow cytometry dot plots are shown for each tissue type to demonstrate gating strategy to identify CD45^+^ cells. (B, C, E, F, H, I, K, L, N, O) Representative flow cytometry dot plots of P2RY12 expression on CD45^+^ cells in (B, E, H, K, N) control- and (C, F, I, L, O) PLX5622-treated mice in each tissue. (P) Quantification of percentages and (Q) numbers of P2RY12^+^CD45^+^ cells in the forebrain (FB), hindbrain (HB), blood (Bl), spleen (Sp), and bone marrow (BM). For quantification panels, each symbol represents an individual control (black) or PLX5622 (red)-treated mouse, and bars indicate mean ± SEM. Data shown represent analysis from one experiment with 5 mice per group, repeated in two independent experiments. Statistical significance was calculated using two-way ANOVA with Sidak’s multiple comparisons test. *P < 0.05; **P < 0.01; ****P < 0.0001. (TIF 14813 kb)

Additional file 2: PLX5622 broadly depletes Iba1+ cells. Mice were fed chow containing PLX5622 or control chow for 2 weeks, then microglia depletion was assessed by Iba1 immunohistochemical staining in the (A) hippocampus, (B) cortex, and (C) cerebellum. (D) Quantification of confocal microscopic images for number of Iba1-positive DAPI-positive cells per high-power field (HPF) in the hippocampus (Hpc), cortex (Ctx), and cerebellum (Cb). Three to six images were captured at × 40 per brain region across two separate brain sections for each of four to five independent mice collected in one experiment. Each symbol represents the average number for an individual mouse, with bars indicating mean ± SEM. Statistical significance was calculated using two-way ANOVA with Sidak’s multiple comparisons test, *P* < 0.05; *P < 0.05; **P < 0.01. (TIF 11593 kb)

Additional file 3: PLX5622 treatment does not impact T cell populations in peripheral immune compartments of uninfected mice. Mice were fed PLX5622 or control chow for 2 weeks, then T cell populations were assessed in (A, B, E, F) blood and (C, D, G, H) spleen of uninfected mice. (A, C) Representative flow cytometry plots and example gating strategy to identify CD45^+^ cells. (B, D) Representative flow cytometry plots of CD4 vs CD8 expression on CD45^+^ cells. (E, G) Quantification of percentages and (F, H) total numbers of CD4^+^CD45^+^ vs CD8^+^CD45^+^ cells in uninfected mice. For quantification panels, each symbol represents an individual control (black) or PLX5622 (red)-treated mouse, and bars indicate mean ± SEM. Data shown represent analysis from one experiment with three to five mice per group, repeated in three independent experiments. Multiple unpaired *t* test analyses indicate no significant difference among any of these populations. (TIF 8042 kb)

Additional file 4: PLX5622 treatment does not impact macrophage/monocyte population in peripheral immune compartments of uninfected mice. Mice were fed PLX5622 chow or control chow for 2 weeks, then monocytes/macrophages were assessed in (A–F) blood, (G–L) spleen, and (M–R) bone marrow of uninfected mice. (A, G, M) Representative flow cytometry plots of CD11b expression on CD45^+^-gated cells. (B, H, N) Quantification of percentages and (C, I, O) total numbers of CD11b^+^CD45^+^ cells. (D, J, P) Representative flow cytometry plots of Ly6G vs Ly6C expression on CD11b^+^CD45^+^ cells. (E, K, Q) Quantification of percentages and (F, L, R) total numbers of Ly6G^+^CD45^+^ vs Ly6C^+^CD45^+^ cells. For quantification panels, each symbol represents an individual control (black) or PLX5622 (red)-treated mouse, and bars indicate mean ± SEM. Data shown represent analysis from one experiment with five mice per group, repeated in three independent experiments. Multiple unpaired *t* test analyses indicate no significant difference among any of these populations. (TIF 11595 kb)

Additional file 5: PLX5622 treatment does not enhance BBB permeability. Mice were fed PLX5622 chow or control chow for 2 weeks, then infected via footpad with WNV-NY (10^2^ PFU). BBB permeability was measured by detection of sodium fluorescein accumulation in brain tissue homogenates derived from (A) olfactory bulb, (B) cortex, (C) cerebellum, (D) brainstem, and (E) spinal cord. Data are represented as mean ± SEM of individual mouse values normalized to serum sodium fluorescein concentration. Group means were then normalized to the mean values for uninfected controls. Statistical significance was calculated using two-way ANOVA with Sidak’s multiple comparisons test, indicating significantly different curves, but no significant difference at any 1 day. *P < 0.05; **P < 0.01; (TIF 3314 kb)

Additional file 6: Control and PLX5622-treated CNS tissue are equally permissive to WNV-NY infection. Mice were fed chow containing PLX5622 or control chow for 2 weeks, infected i.c. with 10 PFU WNV-NY, and then monitored for (A) mortality and (B) weight loss. (A) Mice universally die from i.c. infection with WNV-NY regardless of PLX5622 treatment, with no difference in length of survival as calculated by log-rank (Mantel-Cox) test. (B) Weight was measured daily during acute illness as a measure of illness. After death, the last measured weight was carried through to the end of the experiment. Statistically significant greater weight loss was measured in control mice compared with PLX5622-treated mice as calculated by two-way ANOVA with matched values comparing group means without multiple comparisons. (C-I) Tissue viral loads were measured by plaque assay at 2 and 4 dpi in control (black) or PLX5622-treated (red) mice. (J) Serum viral loads as measured by qRT-PCR. For viral titers, each symbol represents an individual control (black) or PLX5622 (red)-treated mouse and bars indicate mean. Dotted line indicates assay limit of detection. Data shown represent analysis from one experiment with five mice per group. Statistical significance was calculated using two-way ANOVA with Sidak’s multiple comparisons test. ns, not significant at *P* < 0.05; *P < 0.05; ****P < 0.0001. (TIF 7958 kb)

Additional file 7: Impact of CSF1R antagonism on APC co-stimulatory expression is tissue-dependent. Mice were fed PLX5622 or control chow for 2 weeks, then infected via f.p. with WNV-NY (100 PFU). At 4 dpi, leukocytes were isolated from blood (A–C) and draining popliteal LNs (D–F). (A) Representative flow cytometry plots of CD86 expression on CD11c^+^CD45^+^-gated cells in the blood. (B) Quantification of percentages and (C) total numbers of CD86^+^CD11c^+^CD45^+^ cells in the blood. (D) Representative flow cytometry plots of CD80 expression on CD11c^+^CD45^+^-gated cells in the pLN. (E) Quantification of percentages and (F) total numbers of CD80^+^CD11c^+^CD45^+^ cells in the blood. For quantification panels, each symbol represents an individual control (black) or PLX5622 (red)-treated mouse, and bars indicate mean ± SEM. Data shown represent analysis from one experiment with four to five mice per group. Statistical significance was calculated using unpaired *t* test. For all data: ns, not significant at *P* < 0.05; *P < 0.05; **P < 0.01. (TIF 4465 kb)

Additional file 8: PLX5622 treatment does not impact T cell activation in spleens of WNV-NS5-E218A infected mice. Mice were fed PLX5622 or control chow for 2 weeks, then infected i.c. with WNV-NS5-E218A (10^4^ PFU). At 8 dpi, splenic leukocytes were isolated and analyzed by flow cytometric analysis. (A) Representative flow cytometry gating strategy for CD45^+^ cells. (B) Representative flow cytometry contour plots of CD4 vs CD8 expression on CD45^+^-gated cells. (C) Quantification of percentages and (D) total numbers of CD4^+^CD45^+^ vs CD8^+^CD45^+^ cells. (E) Representative flow cytometry plots of WNV-specific NS4B^+^ tetramer staining of CD8^+^CD45^+^ cells. (F) Quantification of percentages and (G) total numbers of NS4B^+^CD8^+^CD45^+^ cells. (H) Representative flow cytometry plots of CD69 expression on NS4B^+^CD8^+^CD45^+^ cells. (I) Quantification of percentages and (G) total numbers of CD69^+^NS4B^+^CD8^+^CD45^+^ cells. For quantification panels, each symbol represents an individual control (black) or PLX5622 (red)-treated mouse, and bars indicate mean ± SEM. Data shown represent analysis from one experiment with three to five mice per group. Multiple unpaired *t* test analyses indicate no significant difference among any of these populations. (TIF 7349 kb)

## Abbreviations

APC: Antigen-presenting cell
BBB: Blood brain barrier
CNS: Central nervous system
CSF1R: CSF1 receptor
DPI: Days post-infection
FMO: Fluorescence minus one
PFA: Paraformaldehyde
qRT-PCR: Quantitative RT-PCR
WNV: West Nile virus

## References

[1] Petersen LR, Carson PJ, Biggerstaff BJ, Custer B, Borchardt SM, Busch MP (2013). Estimated cumulative incidence of West Nile virus infection in US adults, 1999-2010. Epidemiol Infect.

[2] Guarner J, Shieh W-J, Hunter S, Paddock CD, Morken T, Campbell GL (2004). Clinicopathologic study and laboratory diagnosis of 23 cases with West Nile virus encephalomyelitis. Hum Pathol.

[3] Armah HB, Wang G, Omalu BI, Tesh RB, Gyure KA, Chute DJ (2007). Systemic distribution of West Nile virus infection: postmortem immunohistochemical study of six cases. Brain Pathol Zurich Switz.

[4] Preliminary Maps & Data for 2017 | West Nile Virus | CDC. 2018. Available from: https://www.cdc.gov/westnile/statsmaps/preliminarymapsdata2017/index.html. [Cited 2018 Jun 6].

[5] Samaan Z, McDermid Vaz S, Bawor M, Potter TH, Eskandarian S, Loeb M (2016). Neuropsychological impact of West Nile virus infection: an extensive neuropsychiatric assessment of 49 cases in Canada. PLoS One.

[6] Madden K (2003). West Nile virus infection and its neurological manifestations. Clin Med Res.

[7] Weatherhead JE, Miller VE, Garcia MN, Hasbun R, Salazar L, Dimachkie MM (2015). Long-term neurological outcomes in West Nile virus-infected patients: an observational study. Am J Trop Med Hyg.

[8] Vasek MJ, Garber C, Dorsey D, Durrant DM, Bollman B, Soung A (2016). A complement-microglial axis drives synapse loss during virus-induced memory impairment. Nature.

[9] Ginhoux F, Greter M, Leboeuf M, Nandi S, See P, Gokhan S (2010). Fate mapping analysis reveals that adult microglia derive from primitive macrophages. Science.

[10] Nimmerjahn A, Kirchhoff F, Helmchen F (2005). Resting microglial cells are highly dynamic surveillants of brain parenchyma in vivo. Science.

[11] Schafer DP, Lehrman EK, Kautzman AG, Koyama R, Mardinly AR, Yamasaki R (2012). Microglia sculpt postnatal neural circuits in an activity and complement-dependent manner. Neuron.

[12] Kielian T (2006). Toll-like receptors in central nervous system glial inflammation and homeostasis. J Neurosci Res.

[13] Jang H, Boltz D, McClaren J, Pani AK, Smeyne M, Korff A (2012). Inflammatory effects of highly pathogenic H5N1 influenza virus infection in the CNS of mice. J Neurosci.

[14] Mishra MK, Basu A (2008). Minocycline neuroprotects, reduces microglial activation, inhibits caspase 3 induction, and viral replication following Japanese encephalitis. J Neurochem.

[15] Ano Y, Sakudo A, Kimata T, Uraki R, Sugiura K, Onodera T (2010). Oxidative damage to neurons caused by the induction of microglial NADPH oxidase in encephalomyocarditis virus infection. Neurosci Lett.

[16] Hu S, Sheng WS, Schachtele SJ, Lokensgard JR (2011). Reactive oxygen species drive herpes simplex virus (HSV)-1-induced proinflammatory cytokine production by murine microglia. J Neuroinflammation.

[17] Das S, Mishra MK, Ghosh J, Basu A (2008). Japanese encephalitis virus infection induces IL-18 and IL-1beta in microglia and astrocytes: correlation with in vitro cytokine responsiveness of glial cells and subsequent neuronal death. J Neuroimmunol.

[18] Quick ED, Leser JS, Clarke P, Tyler KL (2014). Activation of intrinsic immune responses and microglial phagocytosis in an ex vivo spinal cord slice culture model of West Nile virus infection. J Virol.

[19] Durrant DM, Daniels BP, Klein RS (2014). IL-1R1 signaling regulates CXCL12-mediated T cell localization and fate within the central nervous system during West Nile virus encephalitis. J Immunol Baltim Md 1950.

[20] Durrant DM, Daniels BP, Pasieka T, Dorsey D, Klein RS (2015). CCR5 limits cortical viral loads during West Nile virus infection of the central nervous system. J Neuroinflammation.

[21] Klein RS, Lin E, Zhang B, Luster AD, Tollett J, Samuel MA (2005). Neuronal CXCL10 directs CD8+ T-cell recruitment and control of West Nile virus encephalitis. J Virol.

[22] Ben-Nathan D, Huitinga I, Lustig S, Van N R, Kobiler D (1996). West Nile virus neuroinvasion and encephalitis induced by macrophage depletion in mice. Arch Virol.

[23] Lim JK, Obara CJ, Rivollier A, Pletnev AG, Kelsall BL, Murphy PM (2011). Chemokine receptor Ccr2 is critical for monocyte accumulation and survival in West Nile virus encephalitis. J Immunol Baltim Md 1950..

[24] Arjona A, Foellmer HG, Town T, Leng L, McDonald C, Wang T (2007). Abrogation of macrophage migration inhibitory factor decreases West Nile virus lethality by limiting viral neuroinvasion. J Clin Invest.

[25] Wang T, Town T, Alexopoulou L, Anderson JF, Fikrig E, Flavell RA (2004). Toll-like receptor 3 mediates West Nile virus entry into the brain causing lethal encephalitis. Nat Med.

[26] Getts DR, Terry RL, Getts MT, Müller M, Rana S, Deffrasnes C (2012). Targeted blockade in lethal West Nile virus encephalitis indicates a crucial role for very late antigen (VLA)-4-dependent recruitment of nitric oxide-producing macrophages. J Neuroinflammation.

[27] Sitati EM, Diamond MS (2006). CD4+ T-cell responses are required for clearance of West Nile virus from the central nervous system. J Virol.

[28] Brien JD, Uhrlaub JL, Nikolich-Zugich J (2008). West Nile virus-specific CD4 T cells exhibit direct antiviral cytokine secretion and cytotoxicity and are sufficient for antiviral protection. J Immunol Baltim Md.

[29] Douglas MW, Kesson AM, King NJ (1994). CTL recognition of West Nile virus-infected fibroblasts is cell cycle dependent and is associated with virus-induced increases in class I MHC antigen expression. Immunology.

[30] Kulkarni AB, Mullbacher A, Blanden RV (1991). In vitro T-cell proliferative response to the flavivirus, West Nile. Viral Immunol.

[31] Liu Y, Blanden RV, Müllbacher A (1989). Identification of cytolytic lymphocytes in West Nile virus-infected murine central nervous system. J Gen Virol.

[32] Shrestha B, Diamond MS (2004). Role of CD8+ T cells in control of West Nile virus infection. J Virol.

[33] Cecchini MG, Dominguez MG, Mocci S, Wetterwald A, Felix R, Fleisch H (1994). Role of colony stimulating factor-1 in the establishment and regulation of tissue macrophages during postnatal development of the mouse. Dev Camb Engl.

[34] Chitu V, Gokhan Ş, Nandi S, Mehler MF, Stanley ER (2016). Emerging roles for CSF-1 receptor and its ligands in the nervous system. Trends Neurosci.

[35] Nandi S, Gokhan S, Dai X-M, Wei S, Enikolopov G, Lin H (2012). The CSF-1 receptor ligands IL-34 and CSF-1 exhibit distinct developmental brain expression patterns and regulate neural progenitor cell maintenance and maturation. Dev Biol.

[36] Wang Y, Szretter KJ, Vermi W, Gilfillan S, Rossini C, Cella M (2012). IL-34 is a tissue-restricted ligand of CSF1R required for the development of Langerhans cells and microglia. Nat Immunol.

[37] Acharya MM, Green KN, Allen BD, Najafi AR, Syage A, Minasyan H (2016). Elimination of microglia improves cognitive function following cranial irradiation. Sci Rep.

[38] Guglielmetti C, Chou A, Krukowski K, Najac C, Feng X, Riparip L-K (2017). In vivo metabolic imaging of traumatic brain injury. Sci Rep.

[39] Rice RA, Pham J, Lee RJ, Najafi AR, West BL, Green KN (2017). Microglial repopulation resolves inflammation and promotes brain recovery after injury. Glia.

[40] Nissen JC, Thompson KK, West BL, Tsirka SE (2018). Csf1R inhibition attenuates experimental autoimmune encephalomyelitis and promotes recovery. Exp Neurol.

[41] Dagher NN, Najafi AR, Kayala KMN, Elmore MRP, White TE, Medeiros R (2015). Colony-stimulating factor 1 receptor inhibition prevents microglial plaque association and improves cognition in 3xTg-AD mice. J Neuroinflammation.

[42] Ebel GD, Dupuis AP, Ngo K, Nicholas D, Kauffman E, Jones SA (2001). Partial genetic characterization of West Nile virus strains, New York state, 2000. Emerg Infect Dis.

[43] Shi P-Y, Tilgner M, Lo MK, Kent KA, Bernard KA (2002). Infectious cDNA clone of the epidemic West Nile Virus from New York City. J Virol.

[44] Zhou Y, Ray D, Zhao Y, Dong H, Ren S, Li Z (2007). Structure and function of Flavivirus NS5 methyltransferase. J Virol.

[45] Szretter KJ, Daniels BP, Cho H, Gainey MD, Yokoyama WM, Gale M (2012). 2’-O methylation of the viral mRNA cap by West Nile virus evades ifit1-dependent and -independent mechanisms of host restriction in vivo. PLoS Pathog.

[46] Diamond MS, Shrestha B, Marri A, Mahan D, Engle M (2003). B cells and antibody play critical roles in the immediate defense of disseminated infection by West Nile encephalitis virus. J Virol.

[47] Samuel MA, Whitby K, Keller BC, Marri A, Barchet W, Williams BRG (2006). PKR and RNase L contribute to protection against lethal West Nile virus infection by controlling early viral spread in the periphery and replication in neurons. J Virol.

[48] Han J, Harris RA, Zhang X-M. An updated assessment of microglia depletion: current concepts and future directions. Mol Brain. 2017;10:25. 10.1186/s13041-017-0307-x PMC5477141 28629387

[49] Daffis S, Szretter KJ, Schriewer J, Li J, Youn S, Errett J (2010). 2′-O methylation of the viral mRNA cap evades host restriction by IFIT family members. Nature.

[50] Feng X, Jopson TD, Paladini MS, Liu S, West BL, Gupta N (2016). Colony-stimulating factor 1 receptor blockade prevents fractionated whole-brain irradiation-induced memory deficits. J Neuroinflammation.

[51] Cavnar MJ, Zeng S, Kim TS, Sorenson EC, Ocuin LM, Balachandran VP (2013). KIT oncogene inhibition drives intratumoral macrophage M2 polarization. J Exp Med.

[52] Hamilton JA, Achuthan A (2013). Colony stimulating factors and myeloid cell biology in health and disease. Trends Immunol.

[53] Bourne N, Scholle F, Silva MC, Rossi SL, Dewsbury N, Judy B (2007). Early production of type I interferon during West Nile virus infection: role for lymphoid tissues in IRF3-independent interferon production. J Virol.

[54] Olson JK, Girvin AM, Miller SD (2001). Direct activation of innate and antigen-presenting functions of microglia following infection with Theiler’s virus. J Virol.

[55] McCandless EE, Zhang B, Diamond MS, Klein RS (2008). CXCR4 antagonism increases T cell trafficking in the central nervous system and improves survival from West Nile virus encephalitis. Proc Natl Acad Sci U S A.

[56] Lokensgard JR, Cheeran MC-J, Hu S, Gekker G, Peterson PK (2002). Glial cell responses to herpesvirus infections: role in defense and immunopathogenesis. J Infect Dis.

[57] Garber C, Vasek MJ, Vollmer LL, Sun T, Jiang X, Klein RS (2018). Astrocytes decrease adult neurogenesis during virus-induced memory dysfunction via IL-1. Nat Immunol.

[58] Veremeyko T, Starossom S-C, Weiner HL, Ponomarev ED. Detection of microRNAs in microglia by real-time PCR in normal CNS and during neuroinflammation. J Vis Exp JoVE. 2012. 10.3791/4097 PMC3476396 22872097

[59] Muscate F, Stetter N, Schramm C, Schulze Zur Wiesch J, Bosurgi L, Jacobs T. HVEM and CD160: Regulators of Immunopathology During Malaria Blood-Stage. Front Immunol. 2018;9:2611. 10.3389/fimmu.2018.02611 PMC6243049 30483269

[60] Wheeler DL, Sariol A, Meyerholz DK, Perlman S (2018). Microglia are required for protection against lethal coronavirus encephalitis in mice. J Clin Invest.

[61] Seitz S, Clarke P, Tyler KL (2018). Pharmacologic depletion of microglia increases viral load in the brain and enhances mortality in murine models of Flavivirus-induced encephalitis. J Virol.

[62] Zhang M-Z, Yao B, Yang S, Jiang L, Wang S, Fan X (2012). CSF-1 signaling mediates recovery from acute kidney injury. J Clin Invest.

[63] Durrant DM, Robinette ML, Klein RS (2013). IL-1R1 is required for dendritic cell-mediated T cell reactivation within the CNS during West Nile virus encephalitis. J Exp Med.

[64] Duncan DS, Miller SD (2011). CNS expression of B7-H1 regulates pro-inflammatory cytokine production and alters severity of Theiler’s virus-induced demyelinating disease. PLoS One.

[65] Welten SPM, Redeker A, Franken KLMC, Oduro JD, Ossendorp F, Čičin-Šain L, et al. The viral context instructs the redundancy of costimulatory pathways in driving CD8(+) T cell expansion. elife. 2015;4. 10.7554/eLife.07486 PMC4558566 26263500

[66] Yeung AWS, Wu W, Freewan M, Stocker R, King NJC, Thomas SR (2012). Flavivirus infection induces indoleamine 2,3-dioxygenase in human monocyte-derived macrophages via tumor necrosis factor and NF-κB. J Leukoc Biol.

[67] Kong K-F, Wang X, Anderson JF, Fikrig E, Montgomery RR (2008). West Nile virus attenuates activation of primary human macrophages. Viral Immunol.

[68] Samuel MA, Diamond MS (2005). Alpha/beta interferon protects against lethal West Nile virus infection by restricting cellular tropism and enhancing neuronal survival. J Virol.

[69] Shrestha B, Wang T, Samuel MA, Whitby K, Craft J, Fikrig E (2006). Gamma interferon plays a crucial early antiviral role in protection against West Nile virus infection. J Virol.

[70] Tsai T-T, Chen C-L, Lin Y-S, Chang C-P, Tsai C-C, Cheng Y-L (2016). Microglia retard dengue virus-induced acute viral encephalitis. Sci Rep.

[71] Phares TW, Stohlman SA, Hwang M, Min B, Hinton DR, Bergmann CC (2012). CD4 T cells promote CD8 T cell immunity at the priming and effector site during viral encephalitis. J Virol.

[72] Amor S, Peferoen LAN, Vogel DYS, Breur M, van der Valk P, Baker D (2014). Inflammation in neurodegenerative diseases--an update. Immunology.

[73] Dendrou CA, McVean G, Fugger L (2016). Neuroinflammation — using big data to inform clinical practice. Nat Rev Neurol.

[74] Hong S, Beja-Glasser VF, Nfonoyim BM, Frouin A, Li S, Ramakrishnan S (2016). Complement and microglia mediate early synapse loss in Alzheimer mouse models. Science.

[75] Fagan K, Crider A, Ahmed AO, Pillai A (2017). Complement C3 expression is decreased in autism spectrum disorder subjects and contributes to behavioral deficits in rodents. Mol Neuropsychiatry.

[76] Janova H, Arinrad S, Balmuth E, Mitjans M, Hertel J, Habes M (2018). Microglia ablation alleviates myelin-associated catatonic signs in mice. J Clin Invest.

[77] Chan LY, Yim EKF, Choo ABH (2012). Normalized median fluorescence: an alternative flow cytometry analysis method for tracking human embryonic stem cell states during differentiation. Tissue Eng Part C Methods.

