# Supplementary figures and images for: CSF1R antagonism limits local restimulation of antiviral CD8+ T cells during viral encephalitis
Source: J Neuroinflammation. 2019 Jan 31;16:22. doi: 10.1186/s12974-019-1397-4 (PMC6354430; doi:10.1186/s12974-019-1397-4)

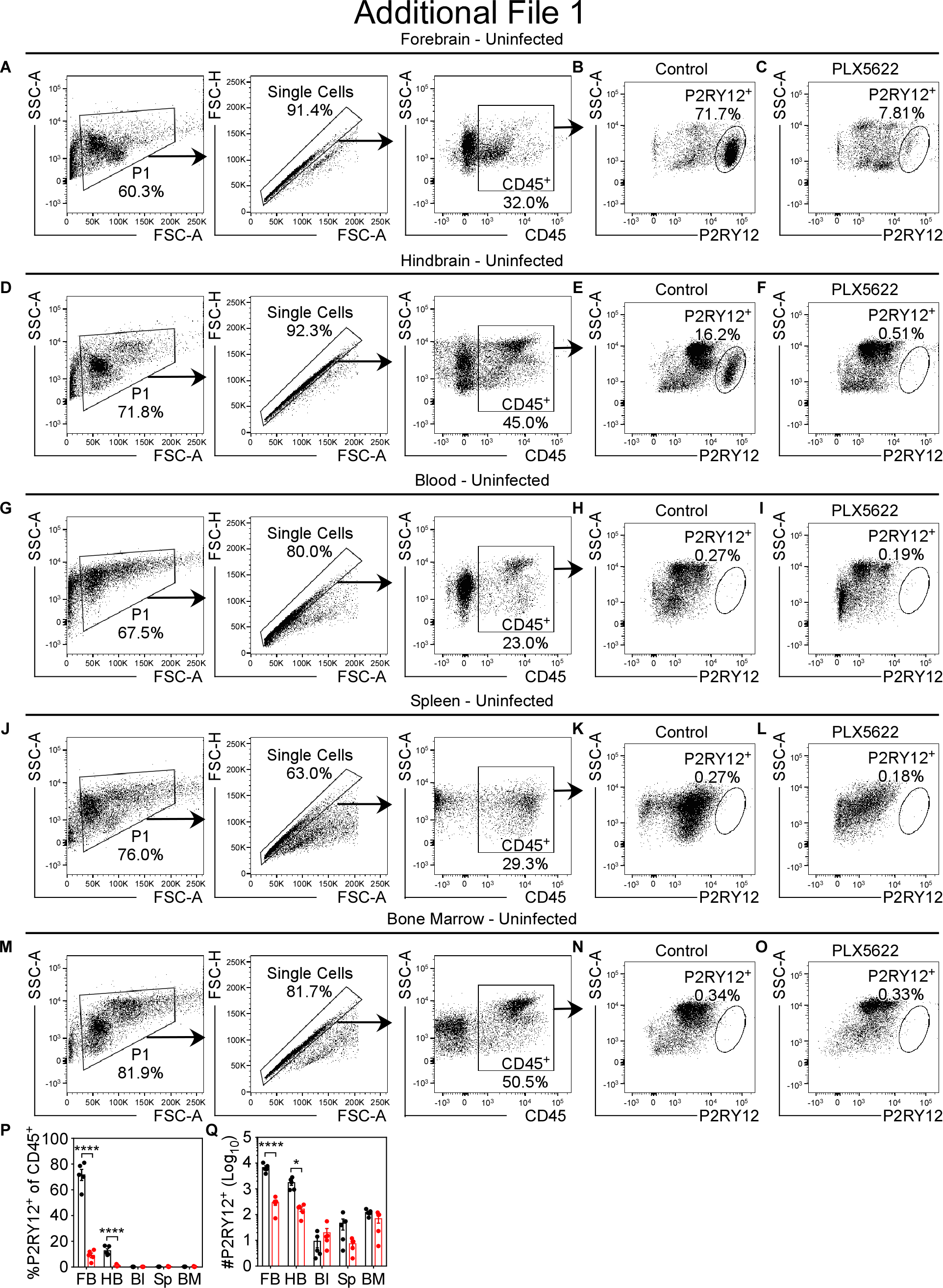

Supplement: Supplementary file 1 — Example gating strategies and P2RY12 staining specificity. Mice were fed chow containing PLX5622 or control chow for 2 weeks, then P2RY12 microglia staining was assessed by flow cytometry in the (A–C) forebrain, (D–F) hindbrain, (G–I) blood, (J–L) spleen, and (M–O) bone marrow of uninfected mice. (A, D, G, J, M) Representative flow cytometry dot plots are shown for each tissue type to demonstrate gating strategy to identify CD45+ cells. (B, C, E, F, H, I, K, L, N, O) Representative flow cytometry dot plots of P2RY12 expression on CD45+ cells in (B, E, H, K, N) control- and (C, F, I, L, O) PLX5622-treated mice in each tissue. (P) Quantification of percentages and (Q) numbers of P2RY12+CD45+ cells in the forebrain (FB), hindbrain (HB), blood (Bl), spleen (Sp), and bone marrow (BM). For quantification panels, each symbol represents an individual control (black) or PLX5622 (red)-treated mouse, and bars indicate mean ± SEM. Data shown represent analysis from one experiment with 5 mice per group, repeated in two independent experiments. Statistical significance was calculated using two-way ANOVA with Sidak’s multiple comparisons test. *P < 0.05; **P < 0.01; ****P < 0.0001. (TIF 14813 kb) [file 12974_2019_1397_MOESM1_ESM.tif]

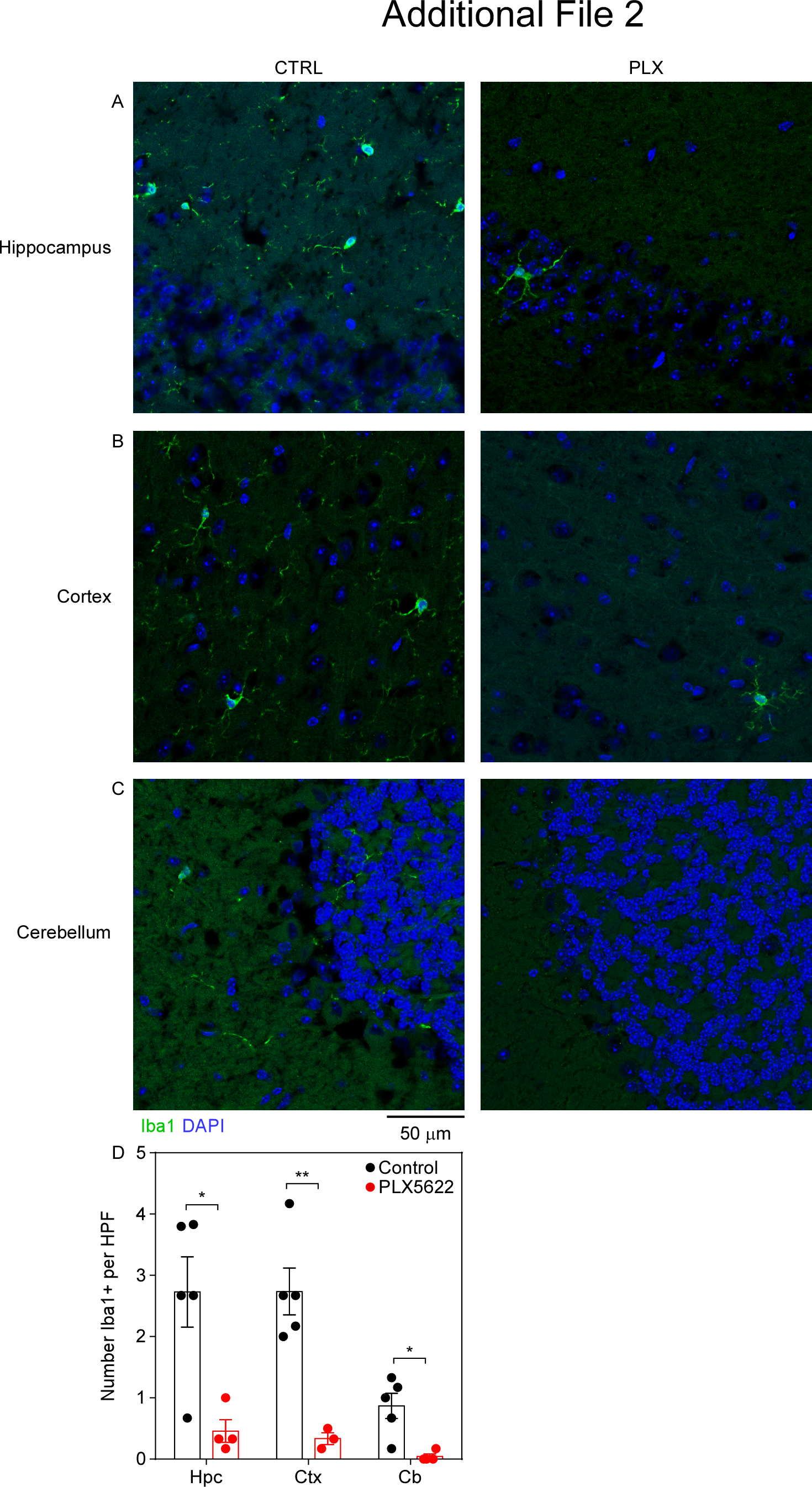

Supplement: Supplementary file 2 — PLX5622 broadly depletes Iba1+ cells. Mice were fed chow containing PLX5622 or control chow for 2 weeks, then microglia depletion was assessed by Iba1 immunohistochemical staining in the (A) hippocampus, (B) cortex, and (C) cerebellum. (D) Quantification of confocal microscopic images for number of Iba1-positive DAPI-positive cells per high-power field (HPF) in the hippocampus (Hpc), cortex (Ctx), and cerebellum (Cb). Three to six images were captured at × 40 per brain region across two separate brain sections for each of four to five independent mice collected in one experiment. Each symbol represents the average number for an individual mouse, with bars indicating mean ± SEM. Statistical significance was calculated using two-way ANOVA with Sidak’s multiple comparisons test, P < 0.05; *P < 0.05; **P < 0.01. (TIF 11593 kb) [file 12974_2019_1397_MOESM2_ESM.tif]

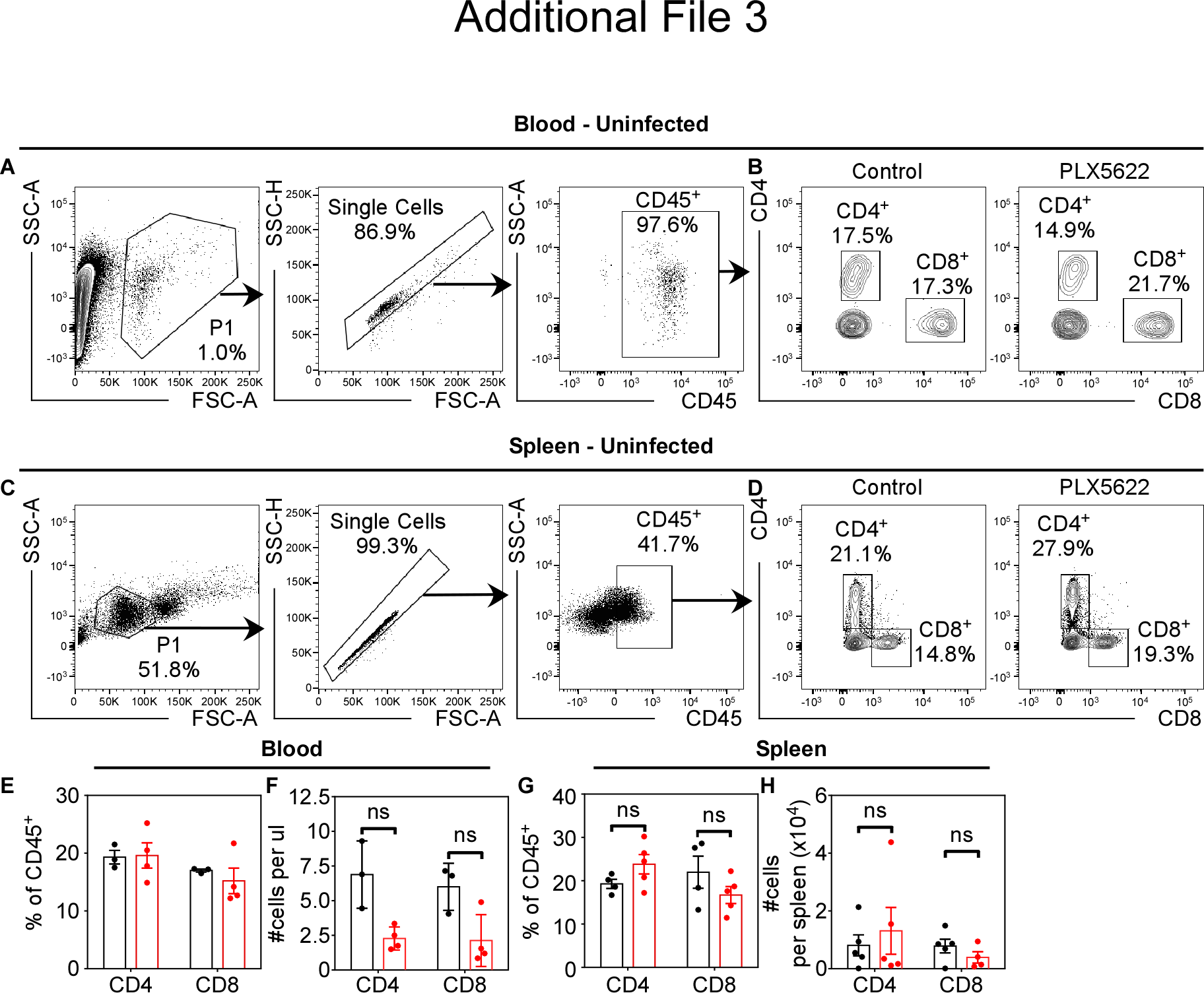

Supplement: Supplementary file 3 — PLX5622 treatment does not impact T cell populations in peripheral immune compartments of uninfected mice. Mice were fed PLX5622 or control chow for 2 weeks, then T cell populations were assessed in (A, B, E, F) blood and (C, D, G, H) spleen of uninfected mice. (A, C) Representative flow cytometry plots and example gating strategy to identify CD45+ cells. (B, D) Representative flow cytometry plots of CD4 vs CD8 expression on CD45+ cells. (E, G) Quantification of percentages and (F, H) total numbers of CD4+CD45+ vs CD8+CD45+ cells in uninfected mice. For quantification panels, each symbol represents an individual control (black) or PLX5622 (red)-treated mouse, and bars indicate mean ± SEM. Data shown represent analysis from one experiment with three to five mice per group, repeated in three independent experiments. Multiple unpaired t test analyses indicate no significant difference among any of these populations. (TIF 8042 kb) [file 12974_2019_1397_MOESM3_ESM.tif]

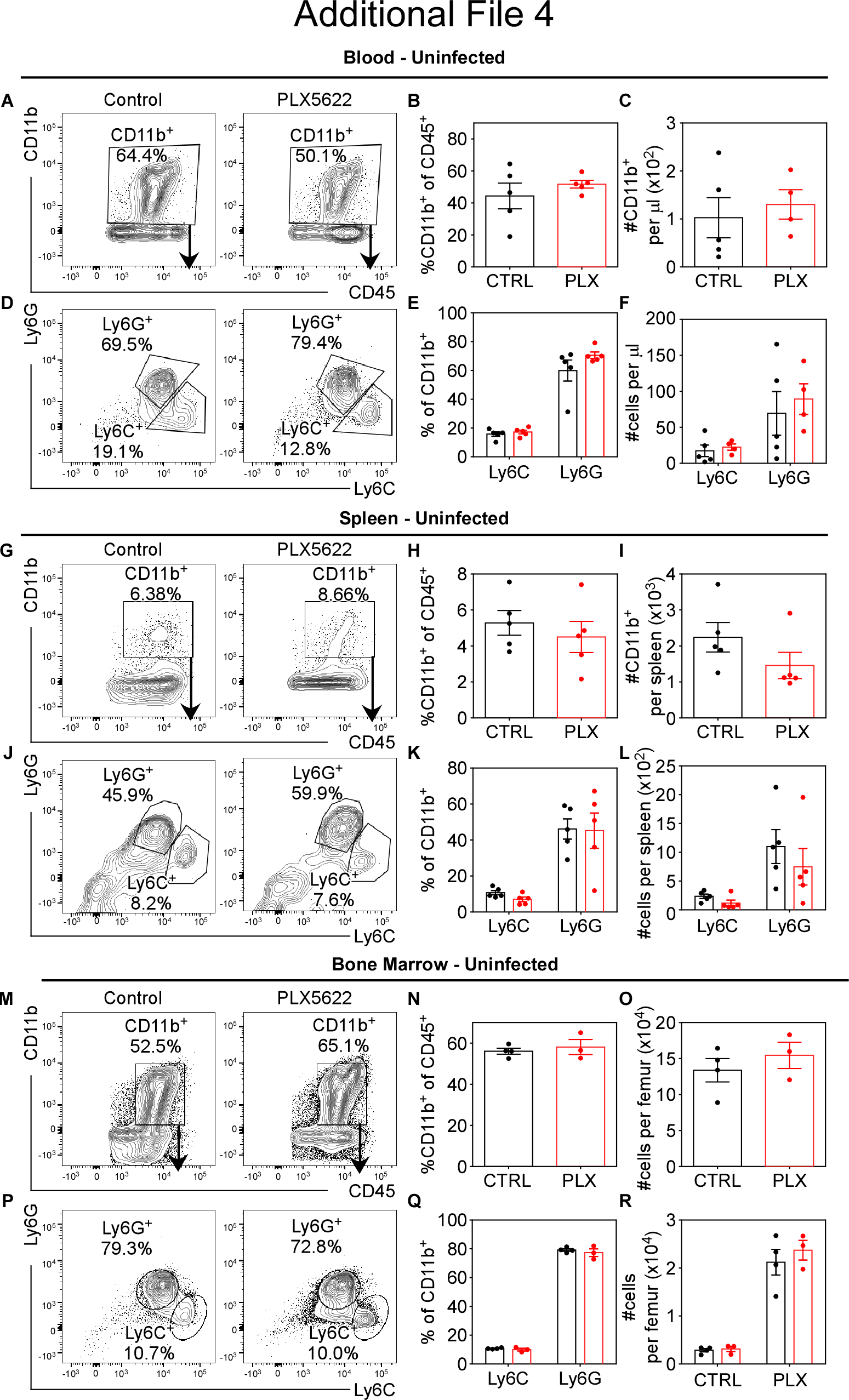

Supplement: Supplementary file 4 — PLX5622 treatment does not impact macrophage/monocyte population in peripheral immune compartments of uninfected mice. Mice were fed PLX5622 chow or control chow for 2 weeks, then monocytes/macrophages were assessed in (A–F) blood, (G–L) spleen, and (M–R) bone marrow of uninfected mice. (A, G, M) Representative flow cytometry plots of CD11b expression on CD45+-gated cells. (B, H, N) Quantification of percentages and (C, I, O) total numbers of CD11b+CD45+ cells. (D, J, P) Representative flow cytometry plots of Ly6G vs Ly6C expression on CD11b+CD45+ cells. (E, K, Q) Quantification of percentages and (F, L, R) total numbers of Ly6G+CD45+ vs Ly6C+CD45+ cells. For quantification panels, each symbol represents an individual control (black) or PLX5622 (red)-treated mouse, and bars indicate mean ± SEM. Data shown represent analysis from one experiment with five mice per group, repeated in three independent experiments. Multiple unpaired t test analyses indicate no significant difference among any of these populations. (TIF 11595 kb) [file 12974_2019_1397_MOESM4_ESM.tif]

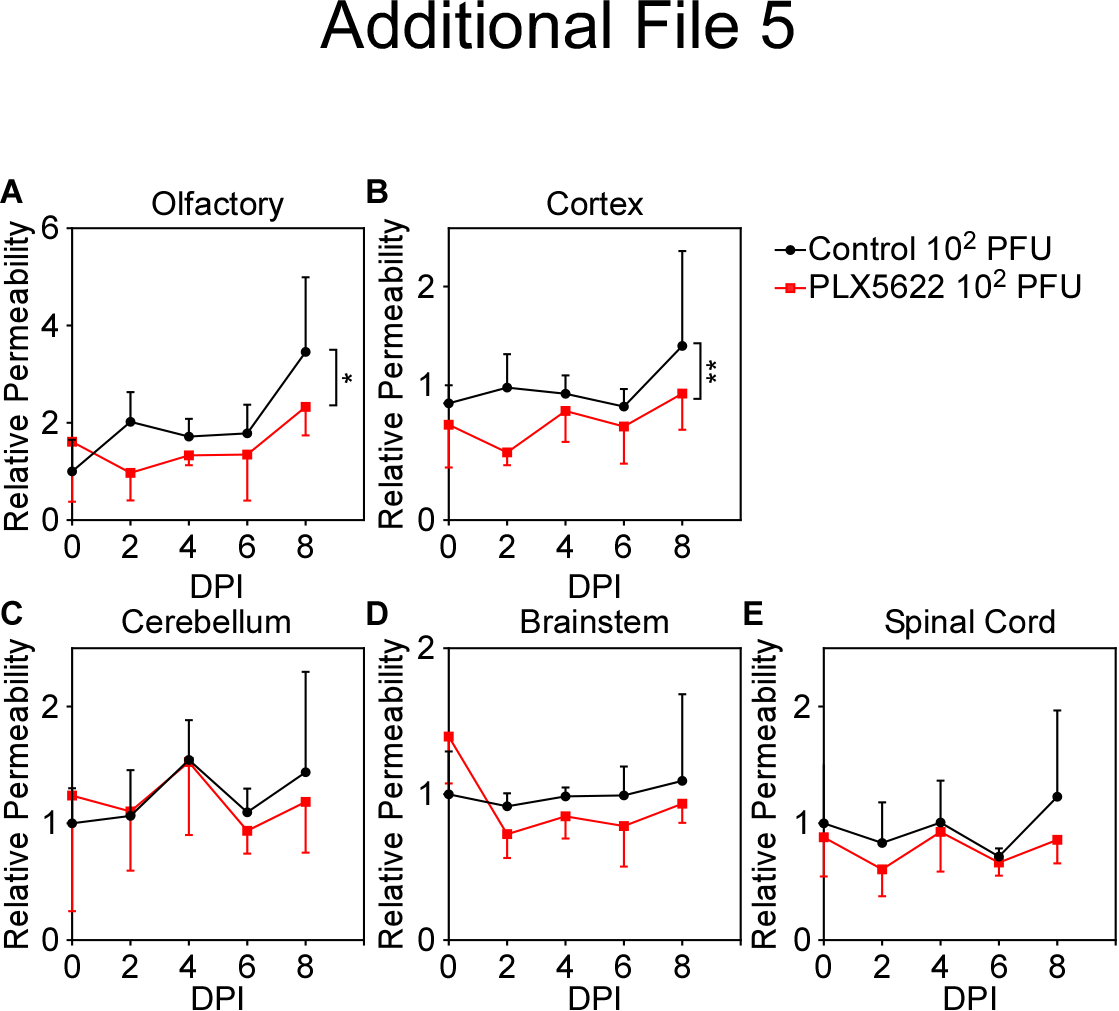

Supplement: Supplementary file 5 — PLX5622 treatment does not enhance BBB permeability. Mice were fed PLX5622 chow or control chow for 2 weeks, then infected via footpad with WNV-NY (102 PFU). BBB permeability was measured by detection of sodium fluorescein accumulation in brain tissue homogenates derived from (A) olfactory bulb, (B) cortex, (C) cerebellum, (D) brainstem, and (E) spinal cord. Data are represented as mean ± SEM of individual mouse values normalized to serum sodium fluorescein concentration. Group means were then normalized to the mean values for uninfected controls. Statistical significance was calculated using two-way ANOVA with Sidak’s multiple comparisons test, indicating significantly different curves, but no significant difference at any 1 day. *P < 0.05; **P < 0.01; (TIF 3314 kb) [file 12974_2019_1397_MOESM5_ESM.tif]

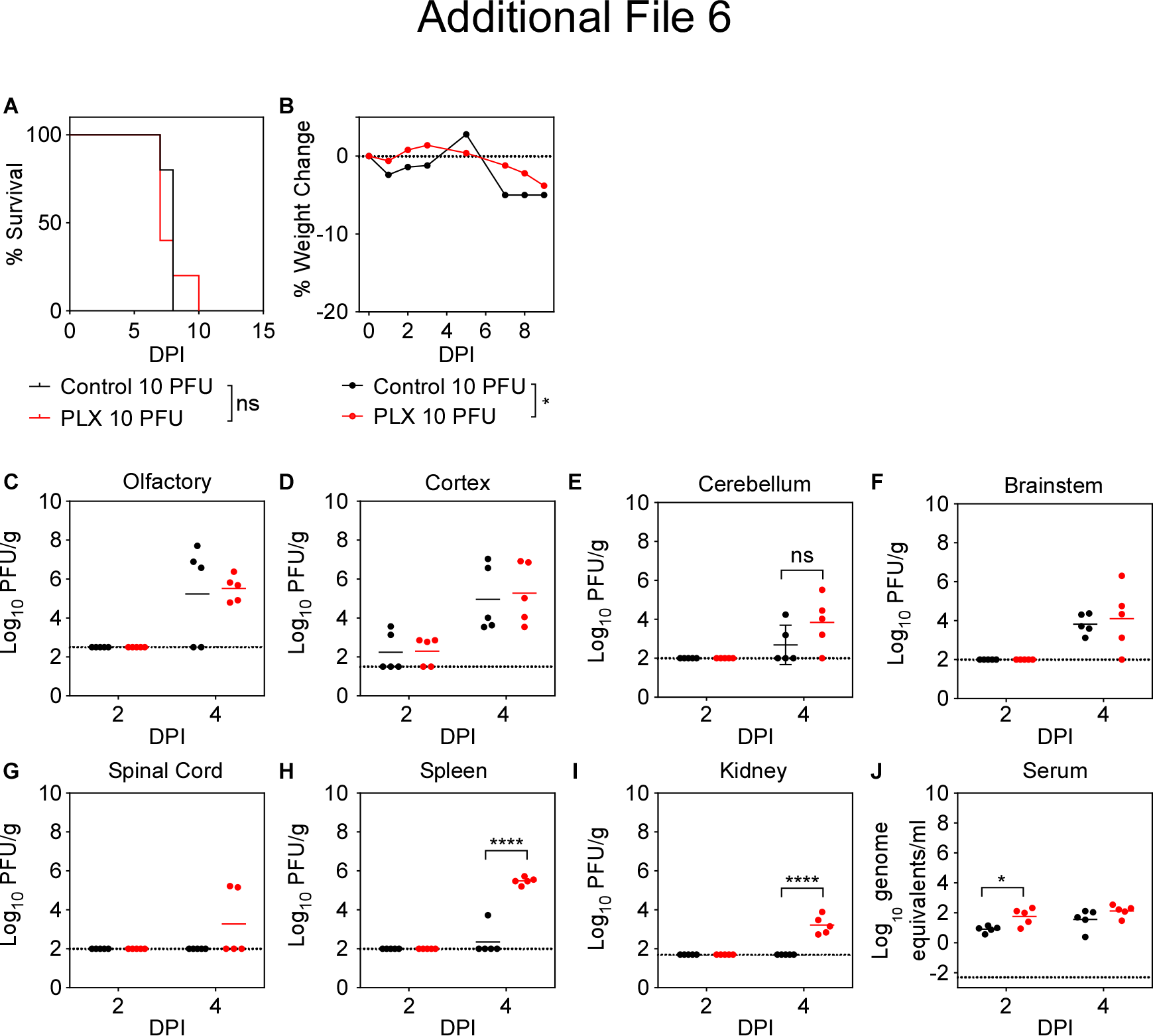

Supplement: Supplementary file 6 — Control and PLX5622-treated CNS tissue are equally permissive to WNV-NY infection. Mice were fed chow containing PLX5622 or control chow for 2 weeks, infected i.c. with 10 PFU WNV-NY, and then monitored for (A) mortality and (B) weight loss. (A) Mice universally die from i.c. infection with WNV-NY regardless of PLX5622 treatment, with no difference in length of survival as calculated by log-rank (Mantel-Cox) test. (B) Weight was measured daily during acute illness as a measure of illness. After death, the last measured weight was carried through to the end of the experiment. Statistically significant greater weight loss was measured in control mice compared with PLX5622-treated mice as calculated by two-way ANOVA with matched values comparing group means without multiple comparisons. (C-I) Tissue viral loads were measured by plaque assay at 2 and 4 dpi in control (black) or PLX5622-treated (red) mice. (J) Serum viral loads as measured by qRT-PCR. For viral titers, each symbol represents an individual control (black) or PLX5622 (red)-treated mouse and bars indicate mean. Dotted line indicates assay limit of detection. Data shown represent analysis from one experiment with five mice per group. Statistical significance was calculated using two-way ANOVA with Sidak’s multiple comparisons test. ns, not significant at P < 0.05; *P < 0.05; ****P < 0.0001. (TIF 7958 kb) [file 12974_2019_1397_MOESM6_ESM.tif]

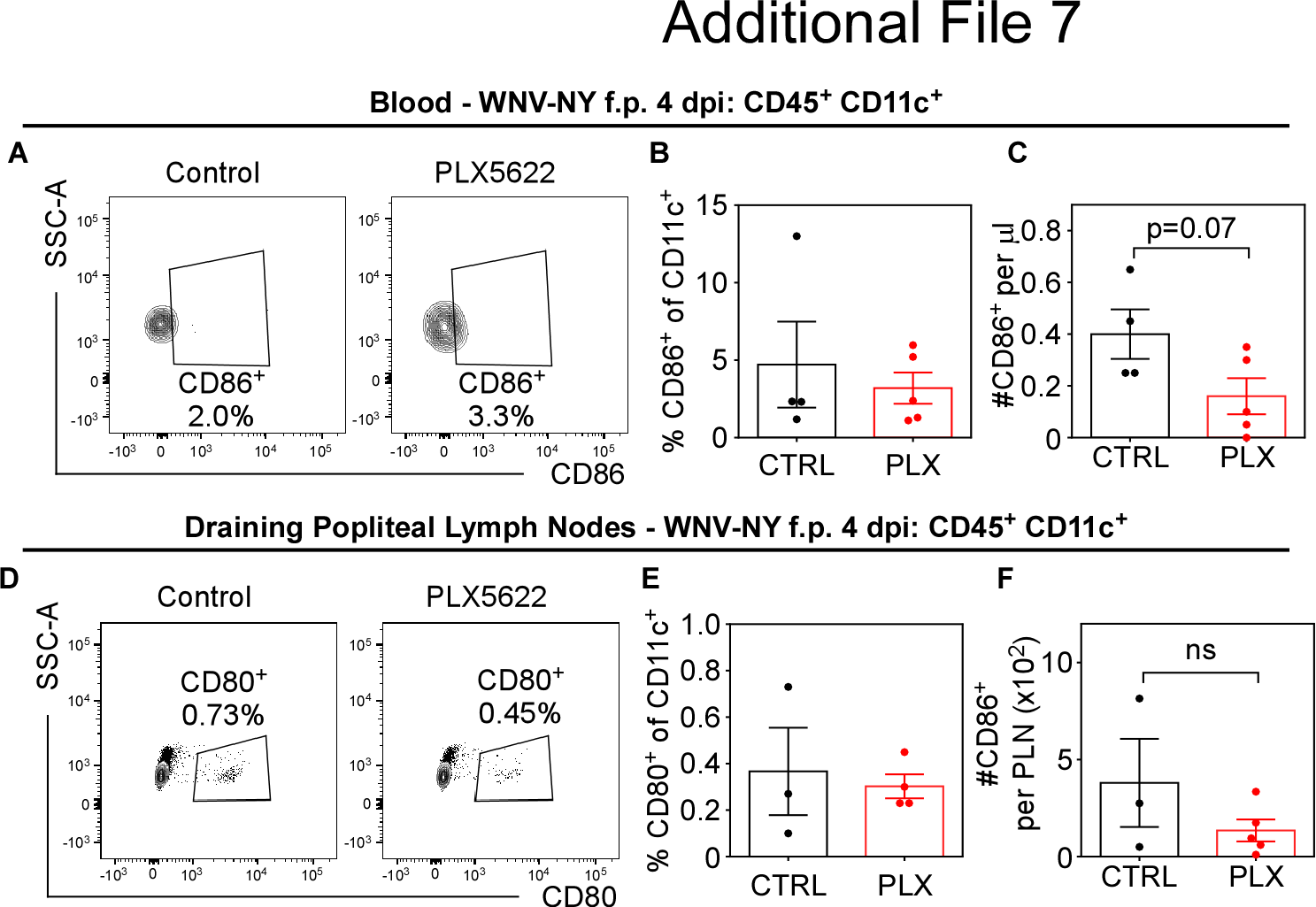

Supplement: Supplementary file 7 — Impact of CSF1R antagonism on APC co-stimulatory expression is tissue-dependent. Mice were fed PLX5622 or control chow for 2 weeks, then infected via f.p. with WNV-NY (100 PFU). At 4 dpi, leukocytes were isolated from blood (A–C) and draining popliteal LNs (D–F). (A) Representative flow cytometry plots of CD86 expression on CD11c+CD45+-gated cells in the blood. (B) Quantification of percentages and (C) total numbers of CD86+CD11c+CD45+ cells in the blood. (D) Representative flow cytometry plots of CD80 expression on CD11c+CD45+-gated cells in the pLN. (E) Quantification of percentages and (F) total numbers of CD80+CD11c+CD45+ cells in the blood. For quantification panels, each symbol represents an individual control (black) or PLX5622 (red)-treated mouse, and bars indicate mean ± SEM. Data shown represent analysis from one experiment with four to five mice per group. Statistical significance was calculated using unpaired t test. For all data: ns, not significant at P < 0.05; *P < 0.05; **P < 0.01. (TIF 4465 kb) [file 12974_2019_1397_MOESM7_ESM.tif]

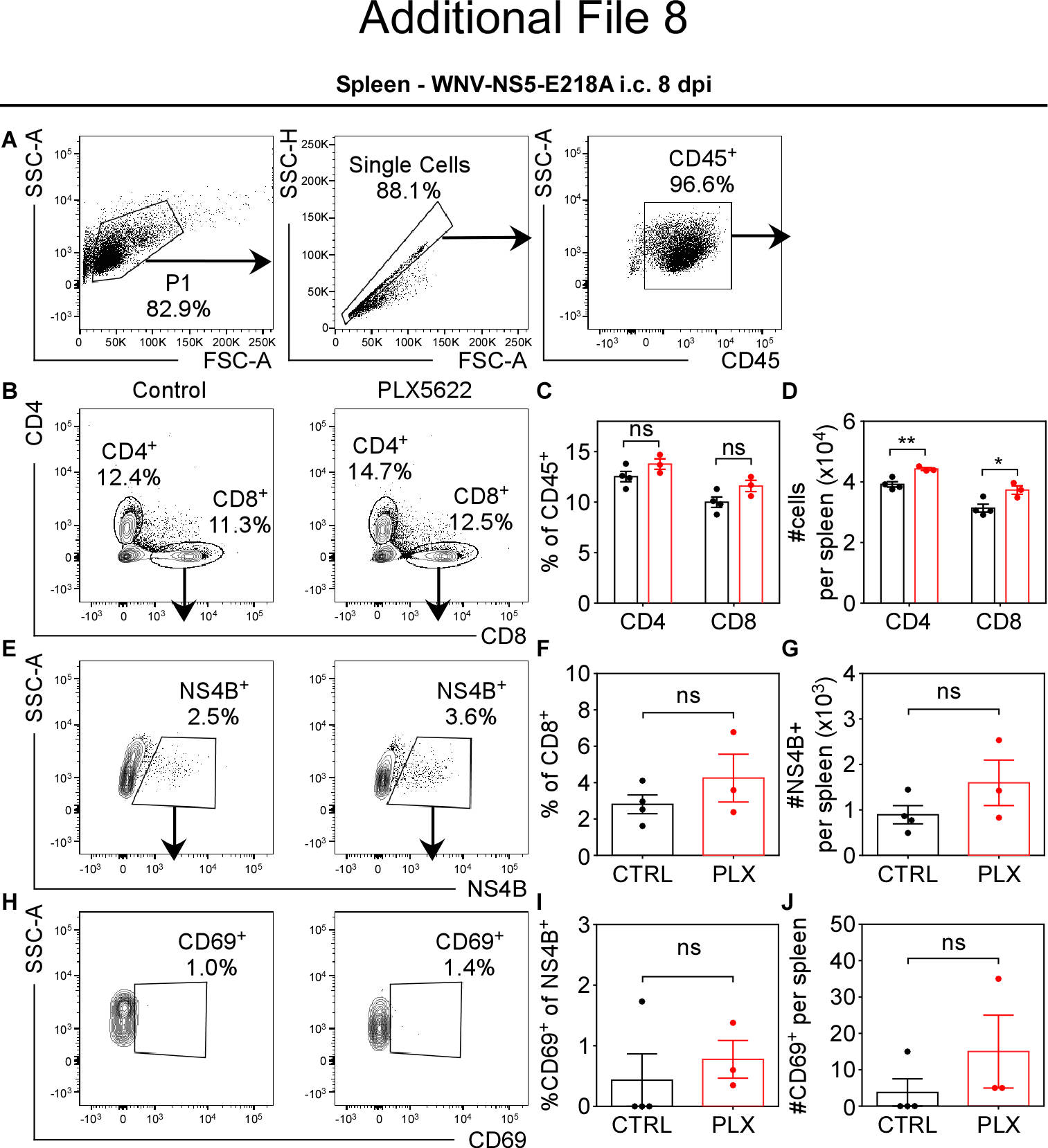

Supplement: Supplementary file 8 — PLX5622 treatment does not impact T cell activation in spleens of WNV-NS5-E218A infected mice. Mice were fed PLX5622 or control chow for 2 weeks, then infected i.c. with WNV-NS5-E218A (104 PFU). At 8 dpi, splenic leukocytes were isolated and analyzed by flow cytometric analysis. (A) Representative flow cytometry gating strategy for CD45+ cells. (B) Representative flow cytometry contour plots of CD4 vs CD8 expression on CD45+-gated cells. (C) Quantification of percentages and (D) total numbers of CD4+CD45+ vs CD8+CD45+ cells. (E) Representative flow cytometry plots of WNV-specific NS4B+ tetramer staining of CD8+CD45+ cells. (F) Quantification of percentages and (G) total numbers of NS4B+CD8+CD45+ cells. (H) Representative flow cytometry plots of CD69 expression on NS4B+CD8+CD45+ cells. (I) Quantification of percentages and (G) total numbers of CD69+NS4B+CD8+CD45+ cells. For quantification panels, each symbol represents an individual control (black) or PLX5622 (red)-treated mouse, and bars indicate mean ± SEM. Data shown represent analysis from one experiment with three to five mice per group. Multiple unpaired t test analyses indicate no significant difference among any of these populations. (TIF 7349 kb) [file 12974_2019_1397_MOESM8_ESM.tif]
